# Heteroatom-bridged molecular belts as containers

**DOI:** 10.1038/s41467-020-17134-3

**Published:** 2020-07-03

**Authors:** Jialin Xie, Xia Li, Shenghua Wang, Anquan Li, Long Jiang, Kelong Zhu

**Affiliations:** School of Chemistry, Sun Yat-Sen University, Guangzhou, 510275 China

**Keywords:** Organic chemistry, Supramolecular chemistry

## Abstract

Hoop-shaped or belt-like molecules have been fascinating not only due to their challenging synthesis, but also unique physical and chemical properties. The incorporation of heteroatoms (N, O, S, etc.) into these belts could alter both molecular structures and electronic properties which will lead to versatile applications, from advanced host-guest systems to functional materials. Despite numerous computational studies, the synthesis and characterization of heteroatom-bridged double-stranded molecular belts remains scarce. Here we report the synthesis, crystal structure, and host-guest chemistry of two novel heteroatom-bridged belt-like macrocycles composed of phenoxathiin. The bowl-shaped belt demonstrates a strong binding affinity (*K*_*a*_ = 3.6 × 10^9^ M^‒2^) towards fullerene C_60_ and forms a 2:1 capsule-like complex with the aid of C‒H···S hydrogen bonds. The column-like belt can bind the cyclic guest [2,2]paracyclophane to form a ring-in-ring complex. The modular synthesis, structural specificity, and diverse host-guest chemistry of cyclophenoxathiins markedly expands the known chemistry of molecular belts.

## Abstract (web summary)

Heteroatom-bearing molecular loops and belts are fascinating but generally difficult to synthesize. Here, the authors demonstrate that O,S-bridged double-stranded molecular belts—cyclophenoxathiins—can be successfully constructed and employed as versatile supramolecular hosts.

## Introduction

Macrocyclic molecules capable of host–guest complexation have been central to the development of modern supramolecular chemistry^1^. A number of well-established families of synthetic macrocycles, including calixarenes^2^, calixpyrroles^3^, resorcinarenes^4^, pillararenes^5^, coronarenes^6^, cyclophanes^7^, and cycloparaphenylenes (CPPs)^8^, have found applications in the fabrication of diverse supramolecular architectures, and have led to advanced systems for sensing^9^, adsorption/separation^10^, drug delivery^11^, and molecular machines^12^. These macrocycles are singly bridged entities—those with repeating units linked via a single-bond bridge.

Recently, double-stranded belt-like macrocycles have attracted significant attention due to their molecular topologies^13^. For example, the cyclization of subunits with double linkages has been shown to result in molecular belts or Möbius strips^14^, and the synthesis of all carbon nanobelts could ultimately enable the bottom-up synthesis of carbon nanotubes^15^. Moreover, belt-like macrocycles can behave as exceptional supramolecular hosts due to their rigid cavities and highly organized internal chemical functionalities. For instance, the intramolecular bridging of resorcinarenes produces cavitands that can be converted into molecular containers and capsules^16^. However, double-stranded belt-like macrocycles are generally difficult to synthesize, especially for highly strained derivatives such as cyclacenes and their analogs^13,17^. One feasible synthetic approach for constructing double-stranded belt-like macrocycles involves the post-synthetic transformation of a singly bridged macrocycle by adding additional chemical bridges. This strategy has been successfully applied to construct cavitands^16^, chrysaoroles^18^, Möbius strips^14^, carbon nanobelts^15^, and, more recently, belt[4]arene[4]tropilidenes^19^. Despite the numerous theoretical studies^20^, there are few reported belt-like macrocycles with double-heteroatom bridges that are constructed via this synthetic method^21,22^. In contrast, this strategy has been successfully applied to synthesis of polymers, including the phenoxathiin-based polyheteroacenes^23^.

**Fig. 1:**
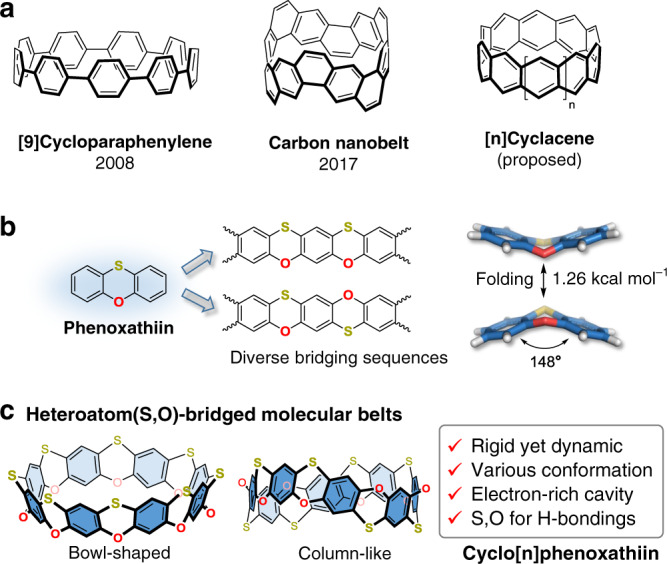
The design of heteroatom-bridged molecular belts. **a** Example of singly bridged macrocycle ([9]cycloparaphenylene), double-stranded molecular belt (carbon nanobelt), and the proposed structure of cyclacenes. **b** The features of phenoxathiin as a building block for constructing molecular belts. **c** The heteroatom(S,O)-bridged molecular belts (cyclo[8]phenoxathiins) report in this work.

Considering the bent structural feature of phenoxathiin^24^, we envision a new class of heteroatom-bridged double-stranded belt-like macrocycles that contain the repeating subunit of phenoxathiin (Fig. 1c). The proposed macrocycle could be thought of as a fused product of oxocalixarenes^2,25^ and thiacalixarenes^2,26^ and defined as cyclo[*n*]phenoxathiins (*n* represents the total number of the phenylenes present in the skeleton of the macrocycle). The highlights of this design include (i) the bent and foldable structure of phenoxathiin (puckering angle ca. 148° and inversion barrier of 1.26 kcal mol^−1^)^24^ allows access to less-strained belt-like macrocycles associated with rigid yet dynamic character to accommodate the adaptive binding of guests, (ii) the electron-donating O- and S-atom linkages not only alter the electronic properties of the macrocycle, but also provide extra interaction sites for hydrogen bonding, and (iii) the alternating bridging sequences of O and S can result in various macrocyclic architectures and nanospaces for diverse host–guest complexation. Herein, we report the first synthesis, structures, and host–guest chemistry of two unique doubly bridged belt-like macrocycles, i.e., cyclo[8]phenoxathiins.

## Results

### Synthesis and characterization

**Fig. 2:**
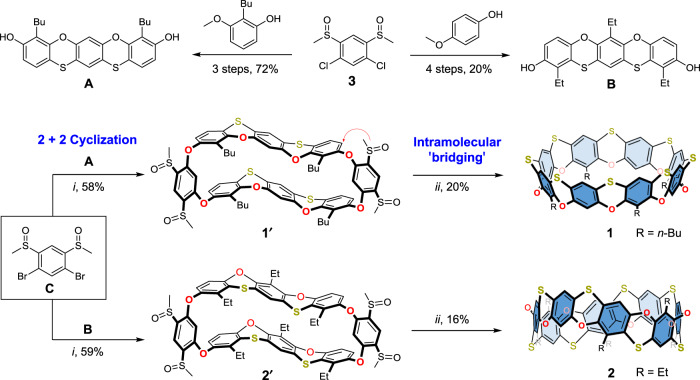
Syntheses of cyclo[8]phenoxathiins 1 and 2 through a cyclization-followed-by-bridging strategy. Conditions: (i) Cs_2_CO_3_, CuI, N,N-dimethylglycine, N,N-dimethylacetamide, 150 °C for 48 h; (ii) CF_3_SO_3_H, 80 °C for 48 h, then pyridine/H_2_O, 105 °C for 15 h. Substituent groups: n-Bu n-butyl, Et ethyl.

A cyclization-followed-by-bridging strategy was adopted to construct cyclo[8]phenoxathiins. This stepwise synthesis allowed us to precisely control the linking sequences of bridging atoms and, consequently, the conformation of the macrocycle. As outlined in Fig. 2, the key steps of the synthesis include a 2 + 2 Ullmann-type coupling cyclization and the subsequent intramolecular Friedel–Crafts reaction with the generated sulfonium cation. The phenoxathiin-derived biphenolic building block **A** can be obtained from monomethylated 2-buylresorcinol and **3** in three steps with a combined yield of 72% (see Supplementary Methods). The butyl groups not only aid the solubility of the final macrocycle, but also eliminate the regioselectivity during the bridging reaction. Replacing the resorcinol with *p*-methoxyphenol during the synthesis provided building block **B** (4 steps, 20%, see Supplementary Methods), which ultimately altered the conformation of the macrocycle formed via 1,4-oxophenylenes. Cyclization of building block **A** or **B** with dibromo **C** afforded the sulfinyl oxo*-*bridged macrocyclic precursor **1′** or **2′** with moderate yields (58% and 59%). Finally, cyclo[8]phenoxathiins **1** and **2** were successfully obtained by carrying out the intramolecular bridging reaction under acidic conditions. Both **1** and **2** were fully characterized by NMR, mass spectrometry, and single-crystal X-ray diffraction analysis.

**Fig. 3:**
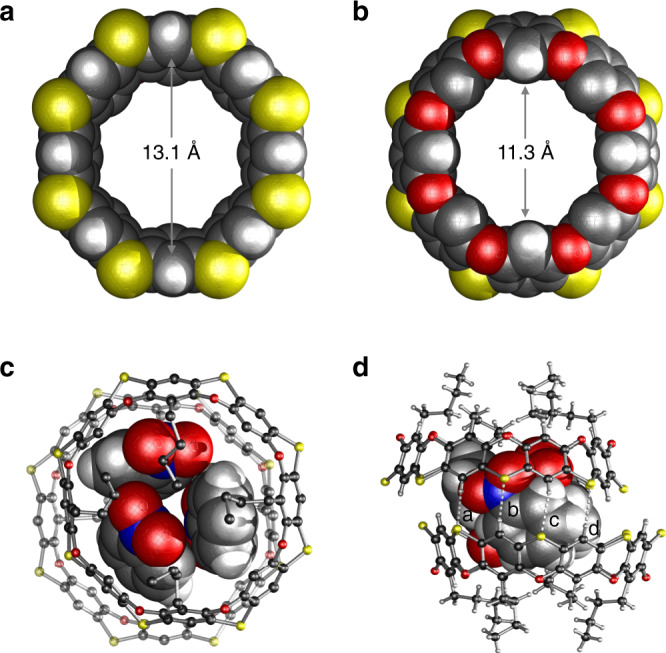
Single-crystal X-ray structure of 1. **a** Space-filling model view from the S rim with the four butyls omitted for clarity. **b** Space-filling model view from the O rim. **c** View of the encapsulated structure of nitrobenzenes in **1** from the top, and (**d**) side view of the complex with hydrogen bonds highlighted. C‒H···S hydrogen bonds: *d*_S···H_ (Å), ∠_C−H···S_ (deg), *d*_C···S_ (Å) −**a**, 2.91, 129, 3.57; **b**, 3.00, 168, 3.92; **c**, 2.97, 165, 3.88; **d**, 3.03, 135, 3.75. *d*_S···H_ = atomic distance between S and H, ∠_C−H···S_ = hydrogen bond angle, *d*_C···S_ = atomic distance between C and S. Color code: S = gold, O = red, N = blue, C = black, H = gray.

**Fig. 4:**
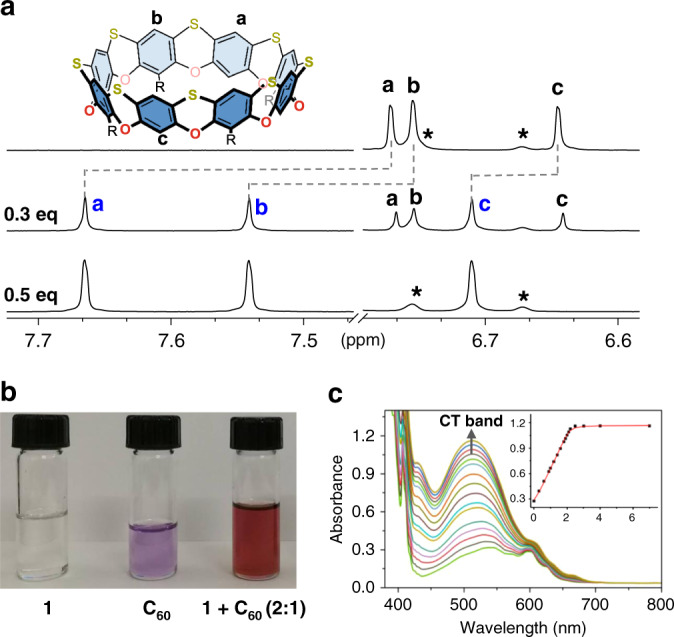
The complexation of 1 with C_60_ in solution. **a** Partial ^1^H NMR spectra of 1.0 mM **1** with different equivalents of fullerene C_60_ in *o*-DCB-*d*_4_ (400 MHz, 298 K). **b** Visualized host–guest interactions between **1** (0.6 mM) and C_60_ (0.3 mM) in *o*-DCB. **c** UV–vis absorption spectra of C_60_ upon titrating with **1** from 0 to 7 equivalents. Inset: titration curves at *λ* = 520 nm. *Residual peaks of deuterated solvent.

Single crystals of **1** were obtained by slow diffusion of ethanol into a nitrobenzene solution of **1**. X-ray crystallography reveals that **1** adopts a bowl-shaped structure that is similar to the structure of cyclodextrins (Fig. 3a, b). Uniquely and as-designed, all eight S atoms are located on the same side of the belt forming the upper rim with a diameter of 13.1 Å (distance from the two opposite carbon atoms across the ring), while the lower rim consists of eight O atoms and has a diameter of 11.3 Å. The dihedral angle of the phenoxathiin unit is ~137° that is smaller than 148° reported for the simplest phenoxathiin^24^. This angular difference indicates that **1** has increased ring strain, which may explain the relatively lower yield in the intramolecular bridging reaction when compared with the linear polymer^23^. Interestingly, in the crystal packing, two of the macrocycles form a dimeric capsule-like structure through weak C‒H∙∙∙S hydrogen bonds from the two upper rims; three nitrobenzene molecules are encapsulated in the well-defined cavity in the dimer (Fig. 3c, d). As shown in Fig. 4a, the ^1^H NMR spectrum of **1** shows three singlets for the aromatic protons, which is consistent with C_4v_ symmetry. The assignment of the peaks was achieved via a combination of 1D and 2D NMR analysis (see Supplementary Information). A m/z peak observed at 1200.1091 on MALDI-TOF-MS analysis further supports the correct structure of **1** (Supplementary Fig. 2). Although the ^1^H NMR spectrum of **1** was found to be concentration-dependent in *o*-dichlorobenzene (*o*-DCB) (Supplementary Fig. 5), the small changes in chemical shifts (0.01 ppm) within the concentration range from 0.020 to 3.0 mM indicated a fairly weak dimerization or self-aggregation of **1**. In addition, a ^1^H diffusion ordered spectroscopy (DOSY)^27^ experiment (Supplementary Fig. 6) gave a diffusion coefficient (D) of 2.15 × 10^–10^ m^2^ s^–1^ at 1.5 mM in *o*-DCB corresponding to a hydrodynamic radius of 7.6 Å. This is consistent with the calculated value of 7.8 Å based on the crystal structure. Thus, the observed encapsulated structure in the solid state is most likely induced by the complexed guest molecule and the crystal packing^28^.

### Host–guest chemistry of 1 with C_60_

Considering that cyclo[8]phenoxathiin **1** has a well-defined bowl-shaped cavity and donor O and S atoms on its peripheral rims, we investigated its ability to behave as a donor host for electron-deficient guests. The cavity size of **1** is comparable to [10]cycloparaphenelene^29^ that is an ideal host for C_60_; based on this observation, we presumed that **1** may have a similar binding ability toward C_60_. After the addition of 0.3 equivalent of C_60_ to **1** in *o*-DCB, the ^1^H NMR spectrum showed two sets of resonances for the protons of **1** indicating a slow-exchange complexation (Fig. 4a). Protons a and b are from the upper rim of **1** shifted downfield by 1.0 and 0.91 ppm, respectively, whereas proton c from the lower rim shifted by 0.08 ppm. Moreover, the ^13^C NMR spectrum of C_60_ in this mixture showed an upfield chemical shift from 142.6 to 140.8 ppm (Supplementary Fig. 8). Upon addition of C_60_ to half an equivalent of **1**, the proton signal of free **1** disappeared. After addition of 7.0 equivalents of C_60_, no further change of the spectrum was observed (Supplementary Fig. 7), indicating a 2:1 binding stoichiometry of **1** with C_60_, which was further supported by MALDI-TOF-MS analysis (Supplementary Fig. 9). An intense m/z peak at 3123.14 corresponding to the C_60_@**1**_2_ complex was observed. The weak intensity of the 1:1 complex signal in the MS analysis and the absence of the signal on the ^1^H NMR spectrum can be ascribed to a fast-kinetic process for the formation of the 1:1 complex^30^.

The complexation between **1** and C_60_ was further evidenced by the visual change of color and a strong charge-transfer band observed at 520 nm on the UV–Vis absorption spectra (Fig. 4b, c). By fitting the UV–Vis titration data with a 2:1 binding mode, the association constants *K*_11_ and *K*_12_ were calculated to be 1.3 × 10^4^ M^–1^ and 2.8 × 10^5^ M^–1^, respectively (Supplementary Fig. 10)^31^. A *K*_12_ ~22 times that of *K*_11_, is indicative of a positive cooperative complexation of C_60_ with **1**. This could be explained by formation of the dimeric structure with fullerenes, which has previously been reported for other macrocycles, including peptide-derived macrocycle^32^, porphyrinoid^33^, and cyclodextrins^34^. The strong binding affinity (*K*_*a*_ = 3.6 × 10^9^ M^‒2^) of **1** toward C_60_ enabled the isolation of the C_60_@**1**_2_ complex directly with silica column chromatography, which could also be useful in the separation and purification of fullerene mixtures.

**Fig. 5:**
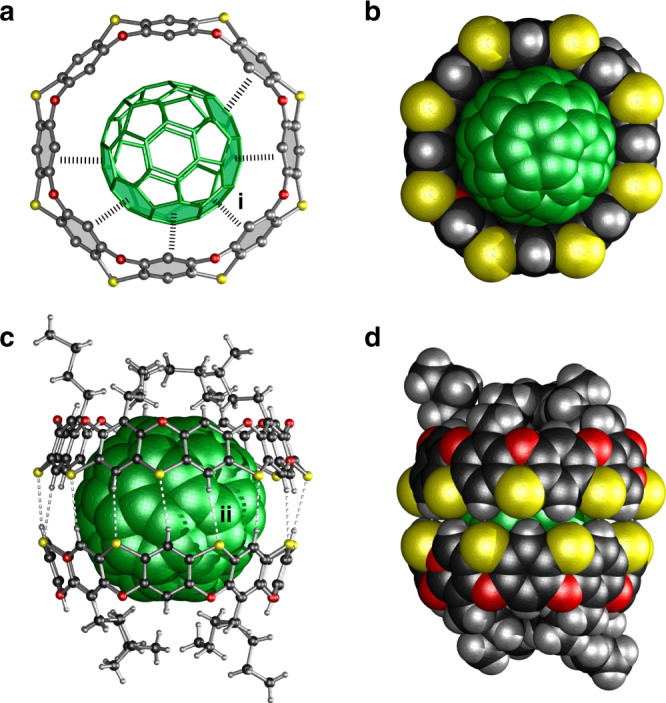
Single-crystal X-ray structure of the complex C_60_@**1**_2_. **a** Hemispherically binded structure with π–π interaction highlighted in bonded dash line. The representative π–π interaction i with a centroid-to-centroid distance of 3.92 Å. **b** Same view in space-filling model. **c** Fully encapsulated structures with C‒H···S hydrogen bonds highlighted in white dash lines. The representative C‒H···S hydrogen bond: *d*_S···H_ (Å), ∠_C−H···S_ (deg), *d*_C···S_ (Å) −ii, 3.00, 166, 3.91. **d** Space-filling model representation of the dimeric structure. Color code: C_60_ = green, S = gold, O = red, C = black, H = gray.

Single crystals of the complex C_60_@**1**_2_ were obtained by slow diffusion of hexane into a 2:1 mixture of **1** and C_60_ in *o*-dichlorobenzene. As shown in Fig. 5a, b, C_60_ resides in the center of the macrocycle in a slightly offset orientation to maximize the π–π interactions with the phenylenes of **1** (centroid-to-centroid distance = 3.92 Å). As mentioned earlier, C_60_ is fully encapsulated in the cavity of the capsule created by the dimerization of two macrocycles through sixteen C‒H∙∙∙S hydrogen bonds from the upper rims (*d*_S···H_ = 3.00 Å, ∠_C−H···S_ = 166°, Fig. 5c, d). This dimerization accounts for the larger downfield chemical shift of protons a and b in the ^1^H NMR spectra and the strong binding affinity.

### Host–guest chemistry of 2 with [2,2]paracyclophane

**Fig. 6:**
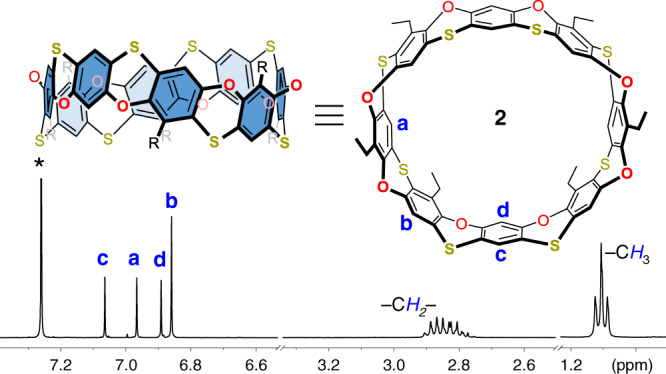
Partial ^1^H NMR spectrum of 2 in chloroform-*d*. Insertion: top and side views of the structure drawing with proton labeling. The four singlets (a, b, c, and d) in the aromatic region indicate the C_2v_ symmetry of the macrocycle. *Residual peaks of deuterated solvent.

**Fig. 7:**
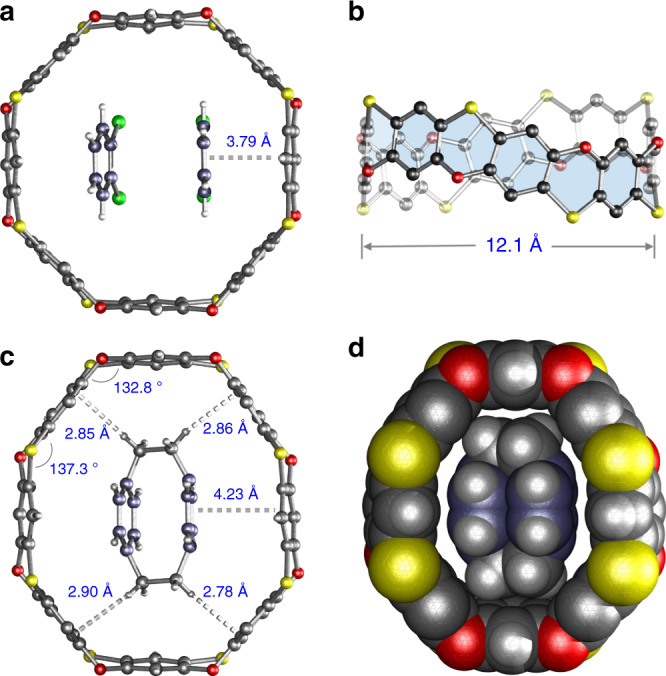
Single-crystal X-ray structures of 2 and [2,2]PCP@2. **a** Ball-and-stick representation of **2** with two *o*-DCBs in the cavity. **b** A view of **2** from front side showing the column-like conformation. **c** Ball-and-stick representation of the ring-in-ring structure of complex [2,2]PCP@**2** with the CH···π and π‒π interaction highlighted in white dash lines. **d** Space-filling model of the ring-in-ring complex. The ethyl groups are omitted for clarity. Color code: S = gold, O = red, Cl = green, C = black or slate-blue, H = white.

Cyclo[8]phenoxathiin **2** was predicted to have a different conformation and cavity size because of the alternative bridging sequences of O and S atoms when compared with **1**. As can be seen from Fig. 6, the ^1^H NMR spectrum of **2** shows four singlets in the aromatic region and two sets of methylene protons, which is consistent with C_2v_ symmetry. The structure was further supported by the observed m/z peak at 1144.0405 from MALDI-TOF-MS analysis (Supplementary Fig. 4). Single crystals of **2** were obtained from slow diffusion of hexanes into a *o*-DCB solution of **2**. As shown in Fig. 7a, 2 adopts an almost column-like octagon-shaped conformation with a distance of 12.1 Å from two parallel phenyl rings. Two *o*-DCB molecules were found to reside in the cavity of **2** to form a 1:2 complex in the solid state. One of the *o*-DCB molecules π-stacks with the phenylene of the wall with a centroid-to-centroid distance of 3.79 Å, while the two *o*-DCBs are almost parallel to each other. Based on this unique 1:2 supramolecular complex, we presumed that cyclo[8]phenoxathiin **2** may exhibit binding affinity toward the cyclic molecule [2,2]paracyclophane ([2,2]PCP), which has a similar molecular structure with two face-to-face phenylenes. The ^1^H NMR spectrum of an equimolar solution of **2** and [2,2]PCP in CDCl_3_ is shown in Supplementary Fig. 11. The signals of protons e and f from [2,2]PCP shifted upfield by 0.01 and 0.02 ppm, respectively, when compared with the free [2,2]PCP, indicating a fairly weak interaction. The changes in the chemical shift could be ascribed to the π–π interaction of [2,2]PCP with the wall of **2**. An association constant of 9 M^–1^ was obtained by fitting the ^1^H NMR titration data with a 1:1 binding mode (Supplementary Figs. 12 and 13).

Although **2** exhibits a weak binding with [2,2]PCP, single crystals of the complex were obtained by slow diffusion of diisopropyl ether into a solution of **2** and [2,2]PCP in chloroform. The guest molecule is disordered over multiple positions due to the weak binding affinity (Supplementary Fig. 15), with one of the favored conformations depicted in Fig. 7c, d. In the complex, **2** is deformed to adopt an oval-like conformation to host [2,2]PCP that results in a ring-in-ring inclusion structure^35,36^. One of the phenylenes of [2,2]PCP is almost parallel to the wall of **2** with a distance of 4.23 Å, indicating a weak π–π interaction. The methylene protons also showed C–H∙∙∙π interaction with the phenylenes, with an average proton to phenyl plane distance of 2.85 Å. These supramolecular interactions might be the main driving forces for formation of the ring-in-ring complex. The deformed octagonal shape of **2** upon complexation is evidence of the rigid yet dynamic nature of cyclophenoxathiin molecular belts.

## Discussion

In summary, we report two novel heteroatom(O,S)-bridged belt-like macrocycles, i.e., cyclo[8]phenoxathiins. Preliminary results reveal them to have different molecular architectures and distinct host–guest chemistry. The bowl-shaped cyclo[8]phenoxathiin **1** demonstrates strong binding affinity toward fullerene C_60_ and forms a 2:1 capsule-like complex. By varying the bridging sequences of the S and O atoms on the two rims, we successfully obtain the other cyclo[8]phenoxathiin **2** with a column-like cavity that can bind [2,2]paracyclophane to form a ring-in-ring inclusion complex. The structural specificity and unique host–guest chemistry of this new class of belt-like macrocycles may give insights into the development of novel host–guest systems and provide further applications in supramolecular chemistry. A comprehensive study of their host–guest chemistry and other physical properties is currently ongoing.

## Methods

### Preparation of cyclo[8]phenoxathiins 1 and 2

Under an N_2_ atmosphere, a 50-mL dry round-bottom flask was charged with compound **1′** (132 mg, 0.099 mmol, 1.0 equiv) and 20 ml of trifluoromethanesulfonic acid. The reaction mixture was stirred at 80 °C for 48 h. After cooling down to room temperature, the reaction mixture was slowly poured into 80 mL of pyridine/ice water = 1/1 (V/V). The reaction mixture was stirred at 105 °C for another 15 h. After cooling down to room temperature, the excess pyridine solvent was removed under reduced pressure and filtered to get a crude product, which was purified by column chromatography on silica gel with dichloromethane/cyclohexane (v/v = 1/5) as eluent to give cyclo[8]phenoxathiin **1** (24 mg, 20% yield) as a white solid. mp > 300 °C (decomp.). ^1^H NMR (400 MHz, 1,1,2,2-tetrachloroethane-*d*_2_) *δ* 7.07 (s, 4H), 6.93 (s, 4H), 6.88 (s, 4H), 2.88 (t, *J* = 7.6 Hz, 8H), 1.50–1.42 (m, 16H), and 0.99 (t, *J* = 6.8 Hz, 12H). ^13^C NMR (100 MHz, 1,1,2,2-tetrachloroethane-*d*_2_) *δ* 153.9, 151.9, 125.6, 123.8, 122.9, 119.8, 118.7, 109.0, 32.0, 23.2, 22.5, 13.9. HRMS (m/z): [M]^+^ calcd. for C_64_H_48_O_8_S_8_, 1200.1109; found: 1200.1091. Cyclo[8]phenoxathiin **2** was synthesized accordingly with a yield of 16%. mp > 300 °C (decomp.). ^1^H NMR (400 MHz, chloroform-*d*) *δ* 7.06 (s, 2H), 6.97 (s, 2H), 6.89 (s, 2H), 6.86 (s, 4H), 2.91–2.75 (m, 12H), and 1.10 (td, *J* = 7.6, 1.2 Hz, 18H). ^13^C NMR (100 MHz, chloroform-*d*) *δ* 154.7, 152.9, 151.3, 149.4, 132.4, 126.2, 125.5, 123.6, 122.5, 120.6, 119.4, 114.9, 109.48, 21.5, 17.4, 14.9, 14.1. HRMS (m/z): [M]^+^ calcd. for C_64_H_40_O_8_S_8_, 1144.0483, found: 1144.0405.

## Supplementary information

Supplementary Information

## Data Availability

All data generated and analyzed during this study are included in this paper and its Supplementary Information files, and are also available from the authors upon reasonable request. Atomic coordinates and structure factors for the reported crystal structures **1**, C_60_@**1**_2_, **2**, and [2.2]PCP@**2** have been deposited in the Cambridge Crystallographic Data Centre (www.ccdc.cam.ac.uk) under accession codes CCDC 1978840-1978843.
