# Supplementary material for: Heteroatom-bridged molecular belts as containers
Source: Nat Commun. 2020 Jul 3;11:3348. doi: 10.1038/s41467-020-17134-3 (PMC7335211; doi:10.1038/s41467-020-17134-3)
Supplement: Supplementary file 1 — Supplementary Information [file 41467_2020_17134_MOESM1_ESM.pdf]

Supplementary Information

Heteroatom-Bridged Molecular Belts as Containers

Xie et al.

Supplementary Methods

Materials and General Methods

2-Butyl-3-methoxyphenol[1] and 1,5-dichloro-2,4-bis(methylsulfinyl)benzene[2] (compound **3**) were synthesized according to literatures. All other reagents were purchased from commercial suppliers and used without further purification unless stated otherwise. Tetrahydrofuran (THF), dichloromethane (DCM) and dimethylacetamide (DMF) were degassed and dried under nitrogen by passing them through a Vigor VSGS-5 Solvent Purification System. Reaction progress was monitored by thin layer chromatography (TLC) or on an Advion Plate Express® Automated TLC plate reader (TLC/CMS). Flash column chromatography was performed over silica gel (200-300 mesh). NMR spectra were recorded on a JEOL 400YH instrument. NMR spectra were internally referenced to tetramethylsilane (^{1}H) or alternatively, to the residual proton solvent signal (^{13}C). All ^{13}C NMR spectra were recorded with complete proton decoupling. UV-vis absorbance spectra were recorded on a Shimadzu UV-2600 spectrophotometer. ESI-MS data were recorded either on an Advion Expression^{L} CMS instrument or a Thermo Fisher Scientific LTQ Orbitrap Elite LC/MS (ESI). MALDI-TOF MS experiments were carried out on a Bruker ultraflex matrix assisted laser desorption-ionization TOF mass spectrometer with DCTB (*trans*-2- [3-(4-*tert*-butylphenyl)-2-methyl-2-propenylidene]malononitrile) as supporting matrix.

Synthesis of [8]cyclophenoxathiin 1

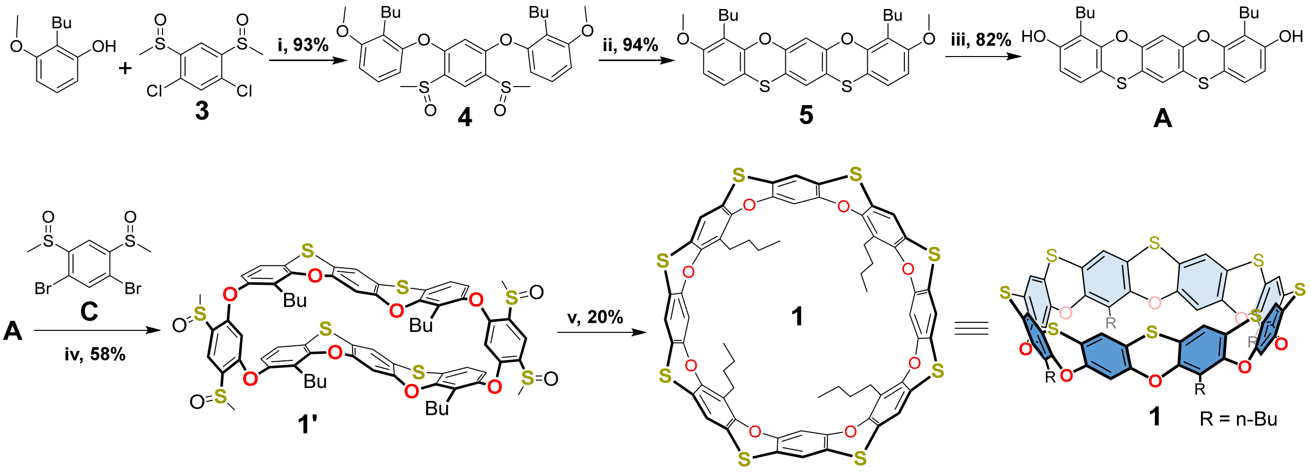

Reaction conditions: i) Cs_2CO_3, DMSO, 120 °C， 26 h; ii) (a) Tf_2O, DCE, RT, 1 h; (b) pyridine, RT, 10 h; (iii) BBr_3, DCM, -15 °C to RT, 20 h; iv) CuI, Cs_2CO_3, *N*,*N*-dimethylglycine, DMAc, 150 °C, 48 h; v) (a) CF_3SO_3H, 80 °C, 48 h; (b) pyridine/H_2O = 1/1 (V/V), 105 °C, 15 h.

Synthesis of [8]cyclophenoxathiin 2

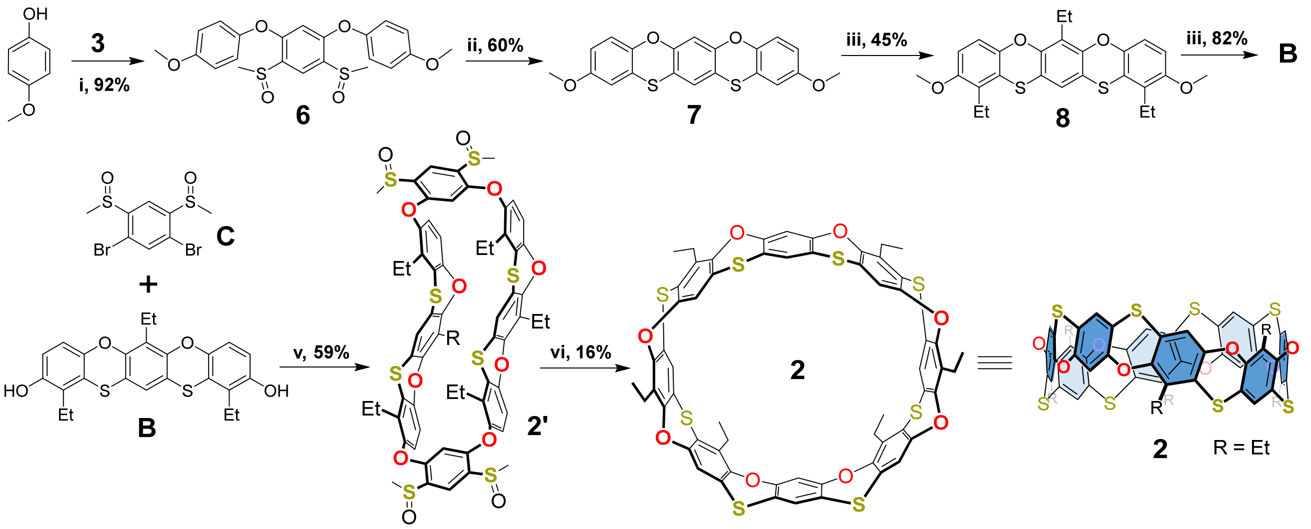

Reaction conditions: i) Cs_2CO_3, DMSO, 120 °C, 26 h; ii) (a)CF_3SO_3H, P_2O_5, RT, 26 h; (b) pyridine/H_2O=1/1 (V/V), 105 °C, 18 h; iii) (a) *n*-BuLi, THF, -5 °C, 1 h; (b) CH_3CH_2I, RT, 24 h; iv) BBr_3, DCM, -10 °C to RT, 20 h; v) CuI, Cs_2CO_3, *N*,*N*-dimethylglycine, DMAc, 150 °C, 48 h; vi) (a) CF_3SO_3H, 80 °C, 48 h; (b) pyridine/H_2O = 1/1 (V/V), 105 °C, 15 h.

3,3'-((4,6-bis(methylsulfinyl)-1,3-phenylene)bis(oxy))bis(2-butyl-1-methoxybenzene) (4)

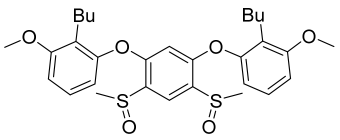

A 250 mL dry Schlenk flask was charged with **3** (3.965 g, 22 mmol, 2.2 equiv), 2-butyl-3-methoxyphenol (2.712 g, 10.1 mmol, 1.0 equiv), Cs_2CO_3 (9.775 g, 30.0 mmol, 3.0 equiv) and anhydrous DMSO (120 mL). Then the reaction mixture was stirred at 120 °C for 26 h under N_2 atmosphere. After cooling down to room temperature, the reaction was quenched by the addition of an aqueous solution of HCl (1 M) and extracted with three portions of CH_2Cl_2. The organic layers were combined and dried over anhydrous Na_2SO_4, filtered, and concentrated in vacuum. The crude product was purified by column chromatography on silica gel with (CH_2Cl_2/CH_3OH = 40/1) as eluent to give the product **4** (5.196 g, 93% yield) as a reddish brown solid. M P: 148-151 °C ^{1}H NMR (400 MHz, Chloroform-*d*) δ 8.54 (s, 0.56 H) and 8.49 (s, 0.44 H), 7.03 (td, J = 8.4, 1.2 Hz, 2H), 6.63 (dt, J = 8.4, 1.2 Hz, 2H), 6.38 (dd, J = 8.4, 0.8 Hz, 2H), 6.05 (s, 1H), 3.79 (s, 6H), 2.89 (s, 2.7H) and 2.86 (s, 3.3 H), 2.49 (td, J = 8.0, 3.2 Hz, 4H), 1.41 – 1.28 (m, 4H), 1.32 – 1.19 (m, 4H), 0.84 (td, J = 7.2, 1.6 Hz, 6H). ^{13}C NMR (100 MHz, Chloroform-*d*) δ 159.1, 156.6, 153.03 and 152.98, 130.4 and 130.2, 127.1, 123.4, 123.2 and 123.1, 111.43 and 111.41, 107.5, 104.6, 55.9, 41.96 and 41.92, 31.7, 23.4, 22.9, 14.1. HRMS (m/z): $[M]^+$ calcd. for $C_{30}H_{39}O_6S_2$, 559.2183, found: 559.2180.

4,8-dibutyl-3,9-dimethoxybenzo[5,6][1,4]oxathiino[3,2-b]phenoxathiine (5)

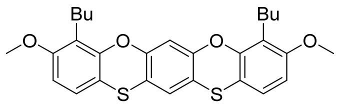

Under an N_2 atmosphere, a dry 500 mL round-bottom flask was charged with **4** (4.263 g, 7.64 mmol, 1.0 equiv) and anhydrous 1,2-dichloroethane (130 mL). Then trifluoromethanesulfonic anhydride (17.202 g, 61.0 mmol, 8.0 equiv) was added in one portion. The reaction mixture was stirred for 1 h at room temperature and then cooled to 0°C in an ice bath, 110 mL pyridine was added drop-wise to the cold solution with stirring. The reaction mixture was subsequently warm to room temperature and stirred for another 10 h. The solvent was removed under reduced pressure and the crude product was purified by column chromatography on silica gel with (petroleum ether/dichloromethane = 3/1) as eluent to give the product **6** (3.548 g, 94% yield) as a white solid. M P: 165-167 °C. ^{1}H NMR (400 MHz, Chloroform-*d*) δ 6.90 (d, J = 8.8 Hz, 2H), 6.88 (s, 1H), 6.77 (s, 1H), 6.57 (d, J = 8.4 Hz, 2H), 3.79 (s, 6H), 2.81 – 2.71 (m, 4H), 1.56 – 1.48 (m, 4H), 1.46 – 1.36 (m, 4H), 0.97 (t, J = 7.2 Hz, 6H). ^{13}C NMR (100 MHz, Chloroform-*d*) δ 157.8, 152.3, 151.0, 124.0, 123.5, 121.6, 117.5, 111.2, 108.1, 106.9, 56.0, 31.8, 23.1, 22.9, 14.2. HRMS (m/z): $[M]^+$ calcd. for $C_{28}H_{31}O_4S_2$, 495.1658, found: 495.1653.

4,8-dibutylbenzo[5,6][1,4]oxathiino[3,2-b]phenoxathiine-3,9-diol (A)

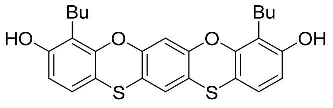

Under an N_2 atmosphere, a dry 500 mL round-bottom flask was charged with compound **5** (2.475 g, 5.01 mmol, 1.0 equiv) and 200 mL anhydrous CH_2Cl_2. Then a CH_2Cl_2 solution (1.0 M) of BBr_3 solution (60 mL, 12.0 equiv) was added drop-wise at -15°C over 10 minutes and stirred for another 30 minutes. The reaction mixture was then slowly warmed to room temperature and stirred for another 20 h. The reaction was quenched by the addition of ice water and extracted with three portions of CH_2Cl_2. The organic layers were combined and dried over anhydrous Na_2SO_4, filtered, and concentrated in vacuum. The crude product was purified by column chromatography on silica gel with (petroleum ether/dichloromethane =1/1) as eluent to give the product **A** (1.910 g, 82% yield) as a white solid. M P: 212-215 °C. ^{1}H NMR (400 MHz, Chloroform-*d*) δ 6.88 (s, 1H), 6.82 (d, J = 8.4 Hz, 2H), 6.76 (s, 1H), 6.51 (d, J = 8.4 Hz, 2H), 4.68 (s, 2H), 2.79 – 2.73 (m, 4H), 1.61 – 1.53 (m, 4H), 1.50 – 1.38 (m, 4H), 0.98 (t, J = 7.2 Hz, 6H). ^{13}C NMR (100 MHz, Chloroform-*d*) δ 153.8, 152.2, 151.3, 124.2, 123.5, 119.5, 117.6, 111.6, 111.1, 107.9, 31.6, 23.2, 22.8, 14.1. HRMS (m/z): $[M]^+$ calcd. for $C_{26}H_{27}O_4S_2$, 467.1345, found: 467.1339.

1,5-dibromo-2,4-bis(methylsulfinyl)benzene (C)

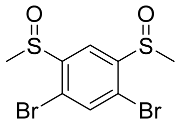

A mixture of 4,6-dibromo-1,3-phenylene-bis(methylsulfane)[3] (4.921 g, 15.0 mmol, 1.0 equiv) and 125 mL glacial acetic acid were charged to a 250 mL round-bottom flask. Then hydrogen peroxide (35%, 3.642 g, 37.5 mmol, 2.5 equiv) was add drop-wise at room temperature and the reaction mixture was stirred for 20 h at room temperature. The glacial acetic acid was removed under vacuum and the residue was dissolved in CH_2Cl_2 and washed several times with water. The organic phase was finally washed with aqueous $NaHCO_3$ solution, dried over $MgSO_4$ and the solvent removed under vacuum. The crude product was purified by column chromatography on silica gel with (CH_2Cl_2/CH_3OH = 40/1) as eluent to give the product **C** (5.023 g, 93% yield) as a white solid. M P: 226-227 °C. ^{1}H NMR (400 MHz, Chloroform-*d*) δ 8.59-8.55 (m, 1H), 7.79 (m, 1H), 2.84–2.87 (m, 6 H). ^{13}C NMR (100 MHz, Chloroform-*d*) δ 148.1, 148.0, 136.6, 136.5, 124.2, 123.9, 121.6, 121.5, 41.8, 41.7. HRMS (m/z): $[M]^+$ calcd. for $C_8H_9Br_2O_2S_2$, 358.8405, found: 358.8399.

Synthesis of Compound 1′

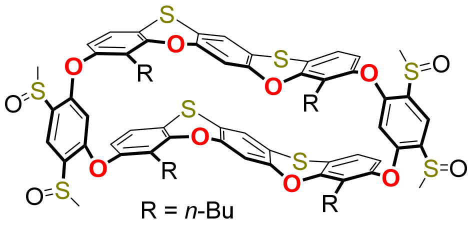

A 100 mL dry Schlenk flask was charged with **A** (260 mg, 0.50 mmol, 1.0 equiv), **C** (180 mg, 0.51 mmol, 1.0 equiv), Cs_2CO_3 (328 mg, 1.0 mmol, 2.0 equiv), CuI (19 mg, 0.1 mmol, 0.2 equiv), *N*,*N*-dimethylglycine (21 mg, 0.2 mmol, 0.4 equiv) and degassed anhydrous dimethylacetamide (40 mL). The reaction mixture was stirred at 150 °C for 48 h under N_2 atmosphere. After cooling down to room temperature, the solvent was removed under reduced pressure and the residue was purified by column chromatography on silica gel with (CH_2Cl_2/CH_3OH = 30/1) as eluent to give the product **1′** (192 mg, 58% yield) as a pale yellow solid. M P > 300 °C (decomp.). ^{1}H NMR (400 MHz, Chloroform-*d*) δ 8.53 – 8.44 (m, 2H), 6.88 – 6.82 (m, 6H), 6.65 – 6.46 (m, 6H), 5.70 – 5.37 (m, 2H), 2.93 – 2.86 (m, 12H), 2.67 – 2.52 (m, 4H), 2.43 – 2.22 (m, 4H), 1.42 – 1.35 (m, 8H), 1.32 – 1.27 (m, 8H), 0.89 – 0.85 (m, 12H). ^{13}C NMR (100 MHz, Chloroform-*d*) δ 157.5 – 156.8 (m), 151.7 – 151.5 (m), 151.3 – 150.8 (m), 129.3 – 127.8 (m), 125.7 – 125.1 (m), 124.8 – 124.4 (m), 123.9 – 123.7 (m), 118.5 – 117.4 (m), 117.2 – 116.0 (m), 107.6, 102.2 – 100.4 (m), 41.8 and 41.7, 32.1 and 32.2, 23.9 and 23.6, 22.8 and 22.6, 14.0 and 13.9. HRMS (m/z): $[M]^+$ calcd. for $C_{68}H_{64}O_{12}S_8$, 1328.2158, found: 1328.2122.

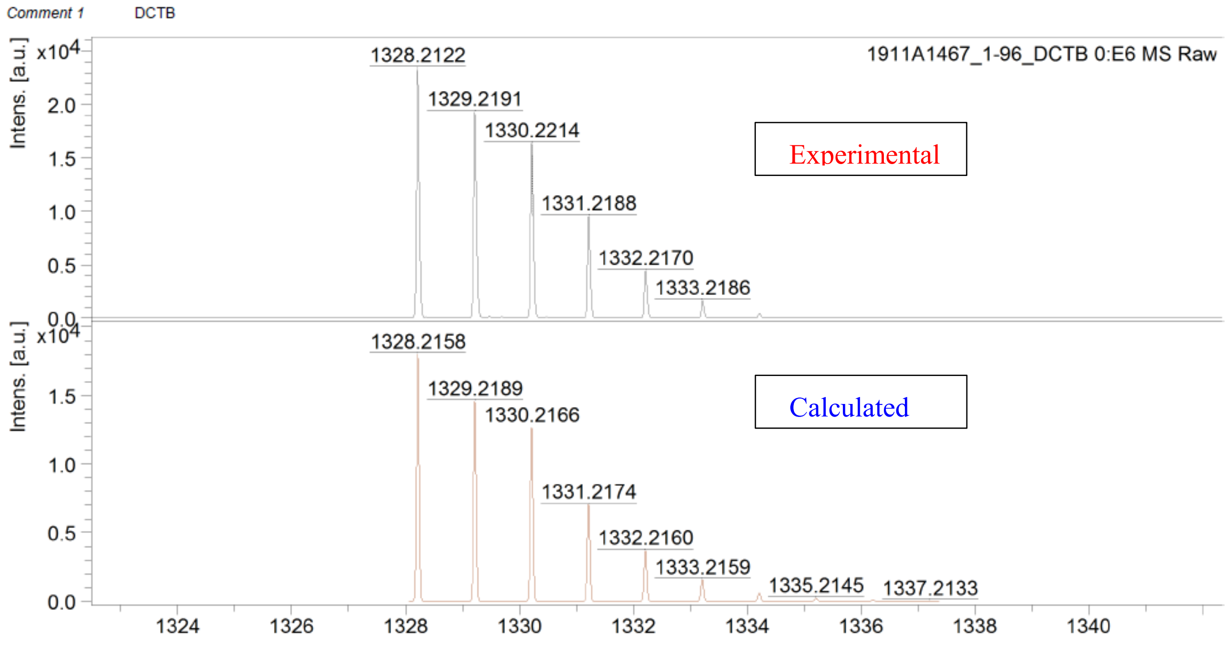

Supplementary Figure 1. MALDI-TOF Mass spectra of compound **1′**.

Synthesis of [8]cyclophenoxathiin 1

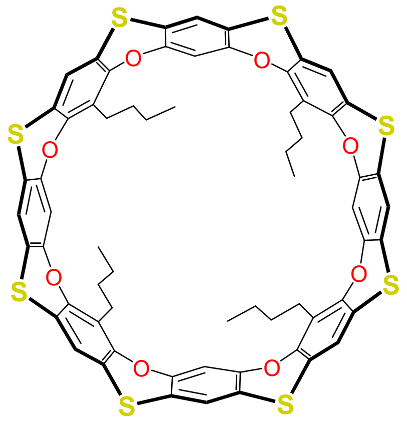

Under an N_2 atmosphere, a 50 mL dry round-bottom flask was charged with compound **1′** (132 mg, 0.099 mmol, 1.0 equiv) and 20 ml trifluoromethanesulfonic acid. The reaction mixture was stirred at 80 ºC for 48 h. After cooling down to room temperature, the reaction mixture was slowly poured into 80 mL pyridine/ice water = 1/1 (V/V). The reaction mixture was stirred at 105 ºC for another 15 hours. After cooling down to room temperature, the excess pyridine solvent was removed under reduced pressure and filtered to get a crude product, which was purified by column chromatography on silica gel with (dichloromethane/cyclohexane = 1/5) as eluent to give the product **1** (24 mg, 20% yield) as a white solid. M P > 300 °C (decomp.). ^{1}H NMR (400 MHz, Tetrachloroethane-d_2) δ 7.07 (s, 4H), 6.93 (s, 4H), 6.88 (s, 4H), 2.88 (t, J = 7.6 Hz, 8H), 1.50–1.42 (m, 16H), 0.99 (t, J = 6.8 Hz, 12H). ^{13}C NMR (100 MHz, Tetrachloroethane-d_2) δ 153.9, 151.9, 125.6, 123.8, 122.9, 119.8, 118.7, 109.0, 32.0, 23.2, 22.5, 13.9. HRMS (m/z): $[M]^+$ calcd. for $C_{64}H_{48}O_8S_8$, 1200.1109, found: 1200.1091.

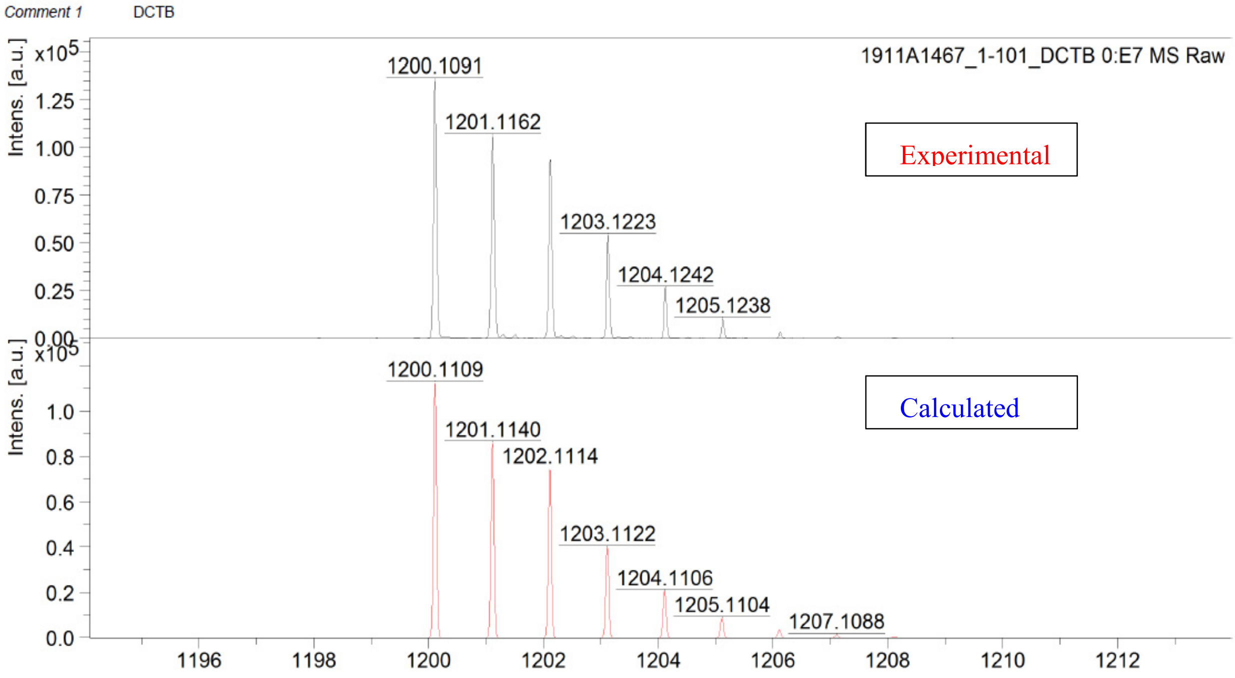

Supplementary Figure 2. MALDI-TOF Mass spectra of compound **1**.

4,4'-((4,6-bis(methylsulfinyl)-1,3-phenylene)bis(oxy))bis(methoxybenzene) (6)

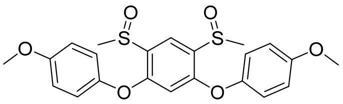

A 250 mL dry Schlenk flask was charged with 4-methoxyphenol (4.097 g, 33.1 mmol, 2.2 equiv), **3** (4.067 g, 15.0 mmol, 1.0 equiv), Cs_2CO_3 (14.662 g, 45.1 mmol, 3.0 equiv) and anhydrous DMSO (130 mL). Then the reaction mixture was stirred at 120 °C for 26 h under N_2 atmosphere. After cooling down to room temperature, the reaction was quenched by the addition of an aqueous solution of HCl (1 M) and extracted with three portions of CH_2Cl_2. The organic layers were combined and dried with anhydrous Na_2SO_4, filtered, and concentrated in vacuum. The crude product was purified by column chromatography on silica gel with (CH_2Cl_2/CH_3OH = 40/1) as eluent to give the product **6** (6.155 g, 92% yield) as a yellow solid. M P: 146-148 °C. ^{1}H NMR (400 MHz, Chloroform-*d*) δ 8.50 (d, J = 2.0 Hz, 0.47H) and 8.45 (d, J = 2.0 Hz, 0.53H), 6.88 – 6.85 (m, 4H), 6.83 – 6.80 (m, 4H), 6.13 (d, J = 1.6 Hz, 1H), 3.77 (d, J = 2.0 Hz, 6H), 2.88 (d, J = 1.6 Hz, 3H), 2.85 (d, J = 1.6 Hz, 3H). ^{13}C NMR (100 MHz, Chloroform-*d*) δ 157.21and 157.18, 157.1, 147.94 and 147.86, 130.4 and 130.2, 123.5 and 123.3, 120.8, 115.2, 104.3 and 104.2, 55.8, 41.9 and 41.8. HRMS (m/z): $[M]^+$ calcd. for $C_{22}H_{23}O_6S_2$, 447.0931, found: 447.0923.

2,10-dimethoxybenzo[5,6][1,4]oxathiino[3,2-b]phenoxathiine (7)

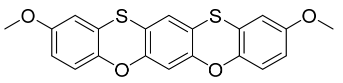

Under an N_2 atmosphere, a 250 mL dry round-bottom flask was charged with compound **6** (1.341 g, 3.0 mmol, 1.0 equiv), P_2O_5 (8.516 g, 60.0 mmol, 20 equiv) and 15 ml anhydrous CH_2Cl_2. Then 15 mL trifluoromethanesulfonic acid was added drop-wise at room temperature and stirred for another 24 h. After cooling down to 0 °C in an ice bath, the reaction was quenched by the addition of 75 mL pyridine/ice water = 1/1 (V/V). The reaction mixture was stirred at 105 °C for another 18 h. After cooling down to room temperature, the excess pyridine was removed under reduced pressure and extracted with three portions of CH_2Cl_2. The organic layers were combined and dried with anhydrous Na_2SO_4, filtered, and concentrated in vacuum. The crude product was purified by column chromatography on silica gel with (petroleum ether / ethyl acetate = 1/4) afforded as eluent to give the product **7** (0.688 g, 60% yield) as a pale yellow solid. M P: 141-144 °C. ^{1}H NMR (400 MHz, Chloroform-*d*) δ 6.91 (d, J = 8.8 Hz, 2H), 6.80 (s, 1H), 6.70 (s, 1H), 6.66 (dd, J = 8.8, 2.8 Hz, 2H), 6.62 (d, J = 2.8 Hz, 2H), 3.75 (s, 6H). ^{13}C NMR (100 MHz, Chloroform-*d*) δ 156.5, 152.3, 145.6, 123.4, 120.6, 118.4, 115.2, 113.3, 111.7, 107.8, 55.8. HRMS (m/z): $[M]^+$ calcd. for $C_{20}H_{15}O_4S_2$, 383.0406, found: 383.0403.

1,6,11-triethyl-2,10-dimethoxybenzo[5,6][1,4]oxathiino[3,2-b]phenoxathiine (8)

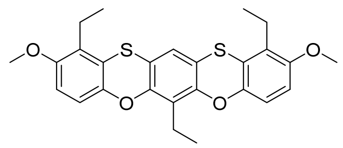

A 100 mL dry Schlenk flask was charged with **7** (765 mg, 2.0 mmol, 1.0 equiv) and 30 mL anhydrous THF. *n*-Butyllithium in *n*-hexane (5 mL, 1.6 M solution, 4.0 equiv) was added drop-wise at –5 ºC over 5 minutes under N_2 atmosphere. The reaction mixture was stirred for 1h, then the CH_3CH_2I (1.092 g, 7.01 mmol, 3.5 equiv) was added at –5 ºC. After 15 minutes the reaction mixture was allowed to warm to room temperature for 24 h and then quenched by the addition of some water and extracted with three portions of EtOAc. The organic layers were combined and dried with anhydrous Na_2SO_4, filtered, and concentrated in vacuum. The crude product was purified by column chromatography on silica gel with (petroleum ether / ethyl acetate = 50/1) as eluent to give the product **8** (419 mg, 45%) as a white solid. M P: 125-127 °C. ^{1}H NMR (400 MHz, Chloroform-*d*) δ 6.88 (d, J = 8.8 Hz, 2H), 6.85 (s, 1H), 6.64 (d, J = 8.8 Hz, 2H), 3.79 (s, 6H), 2.92 (q, J = 7.6 Hz, 2H), 2.71 (q, J = 7.6 Hz, 4H), 1.24 (t, J = 7.6 Hz, 3H), 1.12 (t, J = 7.6 Hz, 6H). ^{13}C NMR (100 MHz, Chloroform-*d*) δ 154.2, 151.0, 146.4, 130.1, 123.4, 122.0, 121.4, 115.9, 115.0, 109.0, 56.1, 20.9, 17.2, 14.5, 13.5. HRMS (m/z): $[M]^+$ calcd. for $C_{26}H_{27}O_4S_2$, 467.1345, found: 467.1341.

1,6,11-triethylbenzo[5,6][1,4]oxathiino[3,2-b]phenoxathiine-2,10-diol (B)

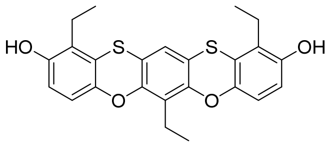

Under an N_2 atmosphere, a dry 250 mL round-bottom flask was charged with compound **8** (1.645 g, 3.53 mmol, 1.0 equiv) and 120 mL anhydrous CH_2Cl_2. Then a CH_2Cl_2 solution (1.0 M) of BBr_3 solution (21 mL, 6.0 equiv) was added drop-wise at -10°C over 10 minutes and stirred for another 30 minutes. The reaction mixture was then slowly warmed to room temperature and stirred for another 20 h. The reaction was quenched by the addition of ice water and extracted with three portions of CH_2Cl_2. The organic layers were combined and dried with anhydrous Na_2SO_4, filtered, and concentrated in vacuum. The crude product was purified by column chromatography on silica gel with (petroleum ether / ethyl acetate =10/1) as eluent to give the product **B** (1.469 g, yield 95%) as a white solid. M P: 176-177 °C. ^{1}H NMR (400 MHz, Chloroform-*d*) δ 6.85 (s, 1H), 6.80 (d, J = 8.4 Hz, 2H), 6.57 (d, J = 8.4 Hz, 2H), 4.59 (s, 2H), 2.89 (q, J = 7.6 Hz, 2H), 2.70 (q, J = 7.6 Hz, 4H), 1.22 (t, J = 7.6 Hz, 3H), 1.17 (t, J = 7.6 Hz, 6H). ^{13}C NMR (100 MHz, Chloroform-*d*) δ 151.1, 150.0, 146.7, 127.6, 123.5, 122.1, 121.4, 115.9, 115.6, 113.7, 20.9, 17.2, 14.4, 13.3. HRMS (m/z): $[M]^+$ calcd. for $C_{24}H_{23}O_4S_2$, 439.1032, found: 439.1030.

Synthesis of Compound 2′

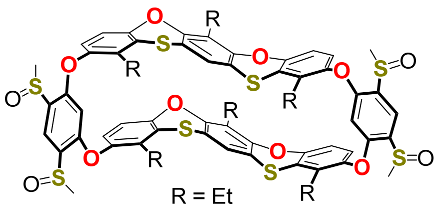

A 100 mL dry Schlenk flask was charged with **B** (219 mg, 0.50 mmol, 1.0 equiv), **C** (180 mg, 0.50 mmol, 1.0 equiv), Cs_2CO_3 (328 mg, 1.0 mmol, 2.0 equiv), CuI (19 mg, 0.1 mmol, 0.2 equiv), *N*,*N*-dimethylglycine (21 mg, 0.2 mmol, 0.4 equiv) and degassed anhydrous dimethylacetamide (40 mL). The reaction mixture was stirred at 150 °C for 48 h under N_2 atmosphere. After cooling down to room temperature, the solvent was removed under reduced pressure and the residue was purified by column chromatography on silica gel with (CH_2Cl_2/CH_3OH = 30/1) as eluent to give the product **2′** (188 mg, 59% yield) as a pale yellow solid. M P > 300 °C (decomp.). ^{1}H NMR (400 MHz, Chloroform-*d*) δ 8.48 – 8.41 (m, 2H), 6.90 – 6.76 (m, 5H), 6.69 – 6.50 (m, 5H), 5.43 – 5.15 (m, 2H), 2.93 – 2.88 (m, 16H), 2.48 – 2.19 (m, 8H), 1.32 – 1.24 (m, 6H), 1.04 – 0.92 (m, 12H). ^{13}C NMR (100 MHz, Chloroform-*d*) δ 158.1 – 157.6 (m), 151.0 – 150.6 (m), 150.5 – 150.0 (m), 148.5 – 147.3 (m), 134.4 – 133.9 (m), 127.6 – 127.3 (m), 124.0 – 123.0 (m), 121.5 – 121.4 (m), 120.8 – 120.0 (m), 116.9 – 115.5 (m), 100.3 – 100.2 (m), 41.8 – 41.6 (m), 32.0 – 29.4 (m), 21.4 – 21.3 (m), 17.4 and 17.2 , 14.5 – 13.7 (m). HRMS (m/z): $[M]^+$ calcd. for $C_{64}H_{56}O_{12}S_8$, 1272.1532, found: 1272.1497.

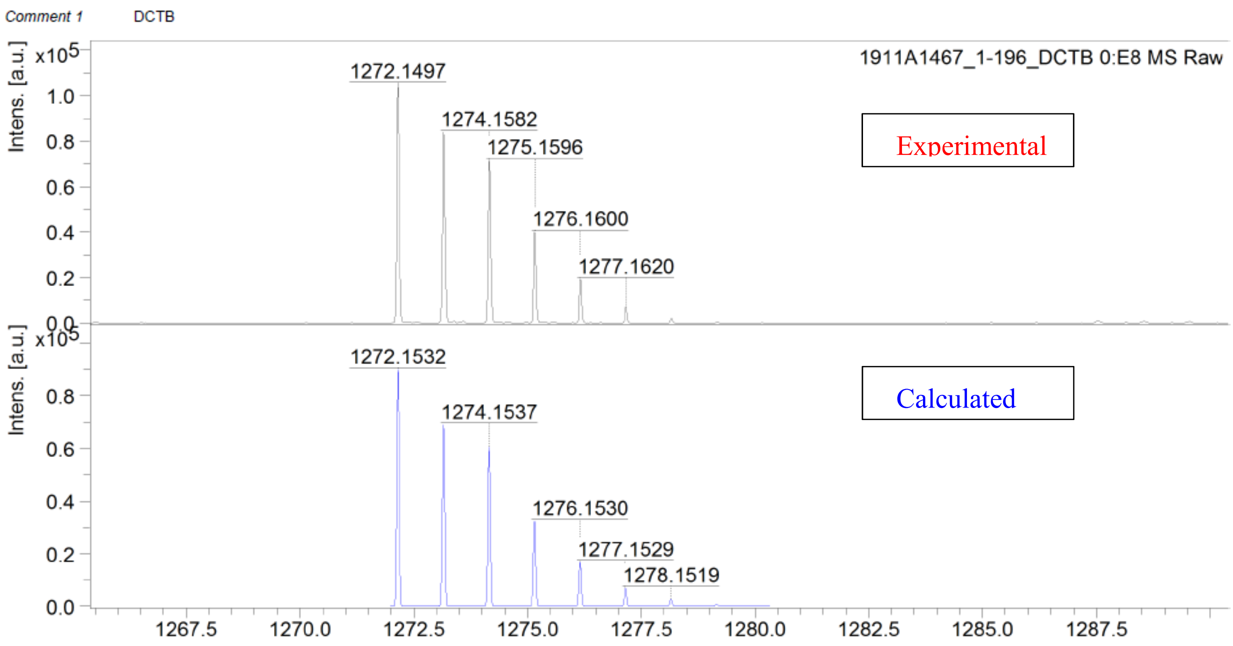

Supplementary Figure 3. MALDI-TOF Mass spectra of compound **2′**.

Synthesis of [8]cyclophenoxathiin 2

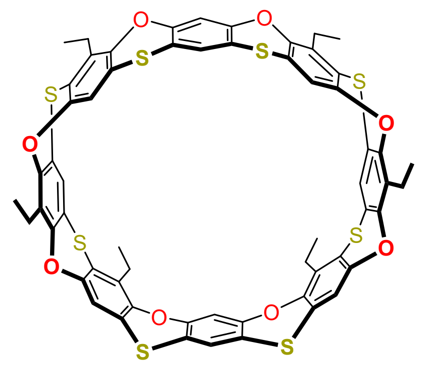

Under an N_2 atmosphere, a 100 mL dry round-bottom flask was charged with compound **2′** (128 mg, 0.099 mmol, 1.0 equiv) and 20 ml trifluoromethanesulfonic acid. The reaction mixture was stirred at 80 °C for 48 h. After cooling down to room temperature, the reaction mixture was slowly poured into 80 mL pyridine/ice water = 1/1 (V/V). The reaction mixture was stirred at 105 °C for another 15 h. After cooling down to room temperature, the excess pyridine solvent was removed under reduced pressure and filtered to get a crude product, which was purified by column chromatography on silica gel with (dichloromethane/cyclohexane = 1/5) as eluent to give the product **2** (19 mg, 16% yield) as a white solid. M P > 300 °C (decomp.). ^{1}H NMR (400 MHz, Chloroform-*d*) δ 7.06 (s, 2H), 6.97 (s, 2H), 6.89 (s, 2H), 6.86 (s, 4H), 2.91 – 2.75 (m, 12H), 1.10 (td, *J* = 7.6, 1.2 Hz, 18H). ^{13}C NMR (100 MHz, Chloroform-*d*) δ 154.7, 152.9, 151.3, 149.4, 132.4, 126.2, 125.5, 123.6, 122.5, 120.6, 119.4, 114.9, 109.48, 21.5, 17.4, 14.9, 14.1. HRMS (m/z): $[M]^+$ calcd. for $C_{64}H_{40}O_8S_8$, 1144.0483, found: 1144.0405.

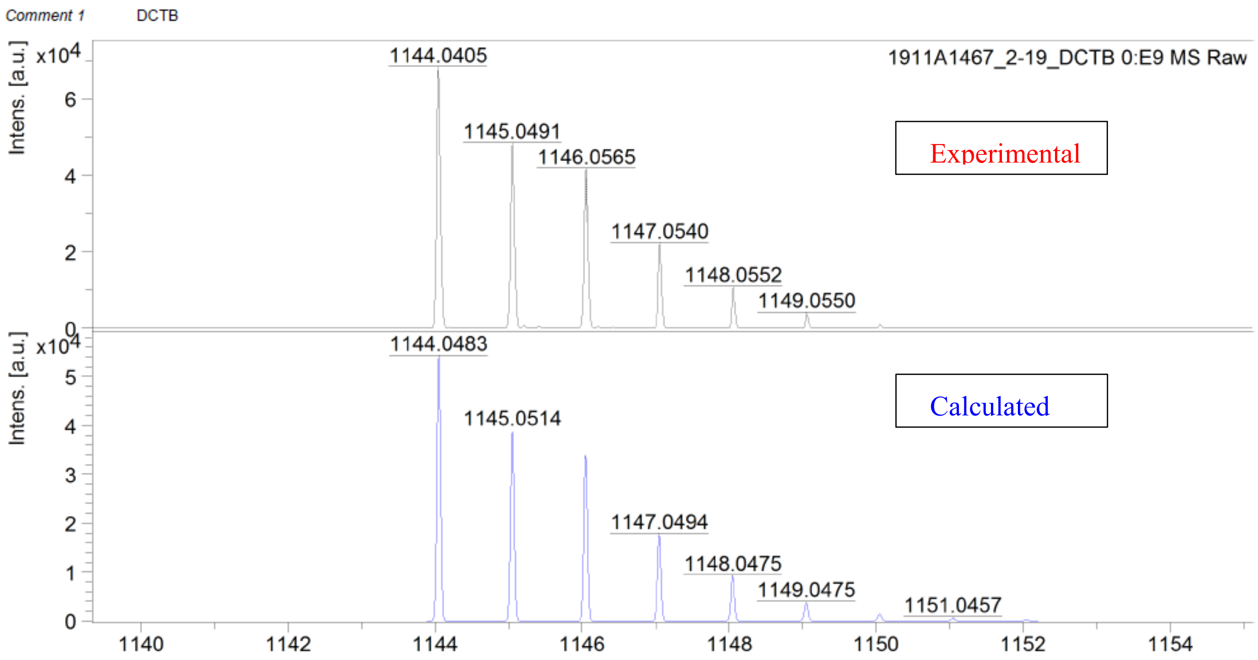

Supplementary Figure 4. MALDI-TOF Mass spectra of compound **2**.

^{1}H NMR Dilution and DOSY Experiment of 1

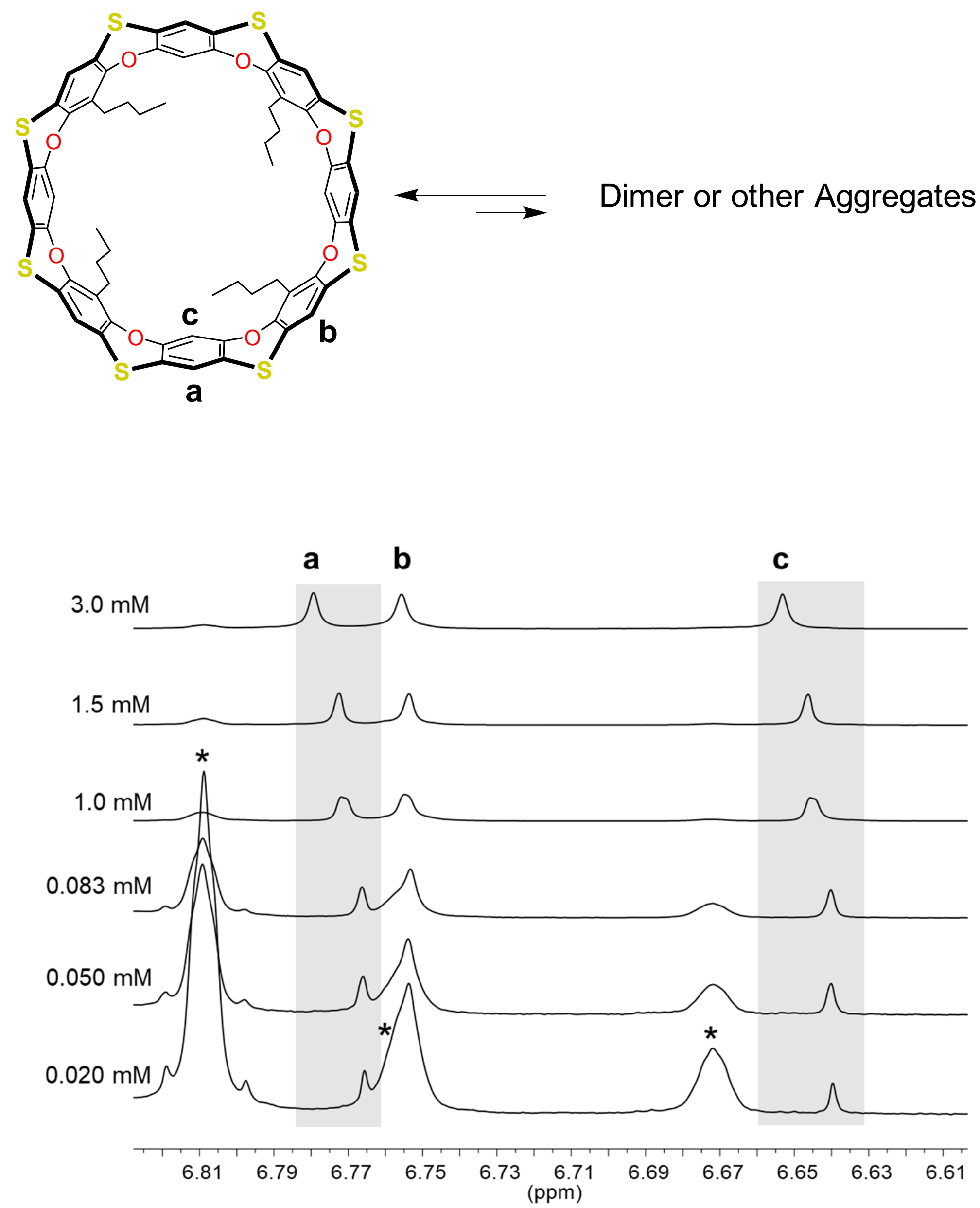

Supplementary Figure 5. ^{1}HNMR dilution experiment of **1** in *o*-dichlorobenzene-*d*4 with concentration from 0.020 mM to 3.0 mM at 298 K. * Residual solvent peak of *o*-DCB-*d*4.

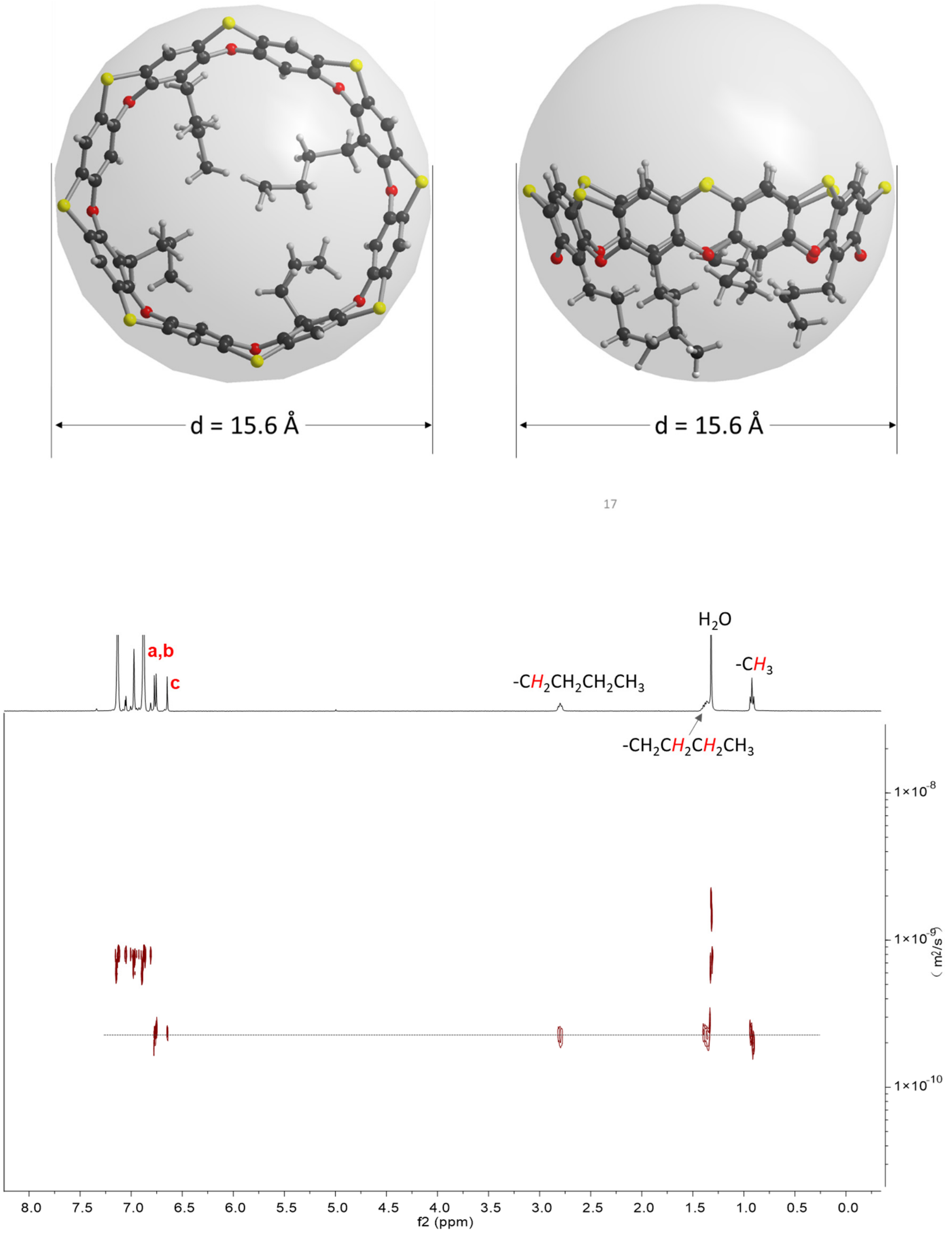

Supplementary Figure 6. Top: structure model of **1** based on the crystal structure. The estimated hydrodynamic radius is about 7.6 Å. Bottom: DOSY experiment of **1** in *o*-DCB-*d*4 (400 MHz, 298K). Diffusion time = 100 ms, Grad 1 = 1 ms, Grad 1 amp = 100 mT/m ~ 0.297 T/m. Relaxation delay = 10 s.

Complexation of C_{60} with 1

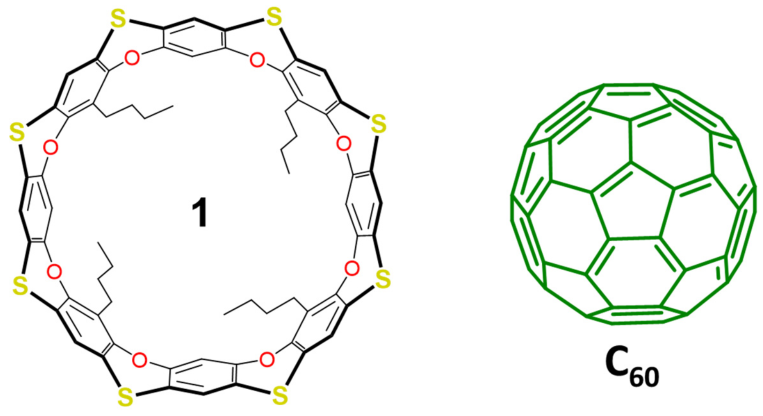

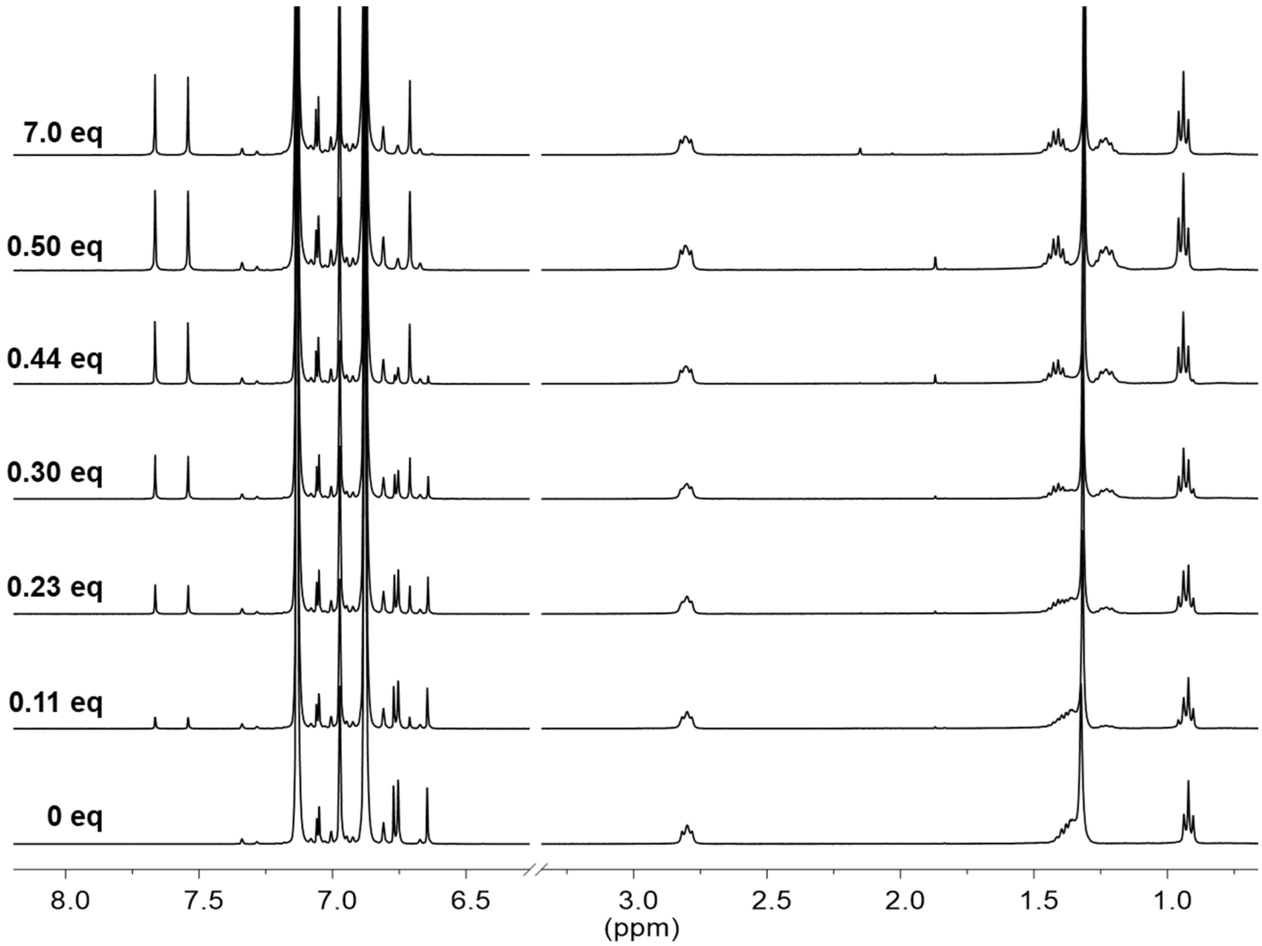

Supplementary Figure 7. NMR titration of **1** with C_{60} from 0 to 7 equivlents (400 MHz, *o*-DCB-d_4, 298 K).

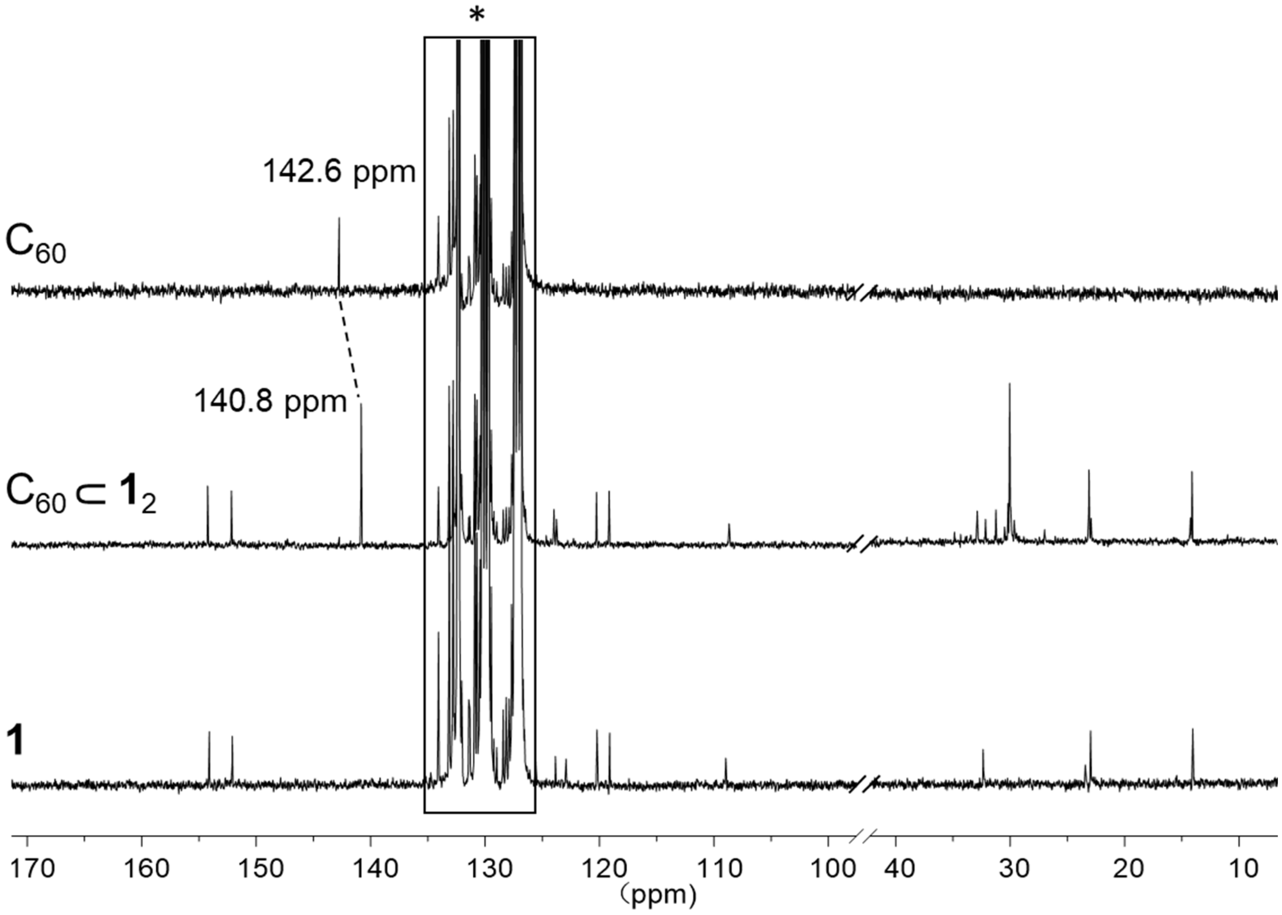

Supplementary Figure 8. Stacked ^{13}C NMR spectra (100 MHz, *o*-DCB-d_4, 298 K) comparison of C_{60}, **1** and the $\mathbf{C_{60}} \subset \mathbf{1}_2$ complex.

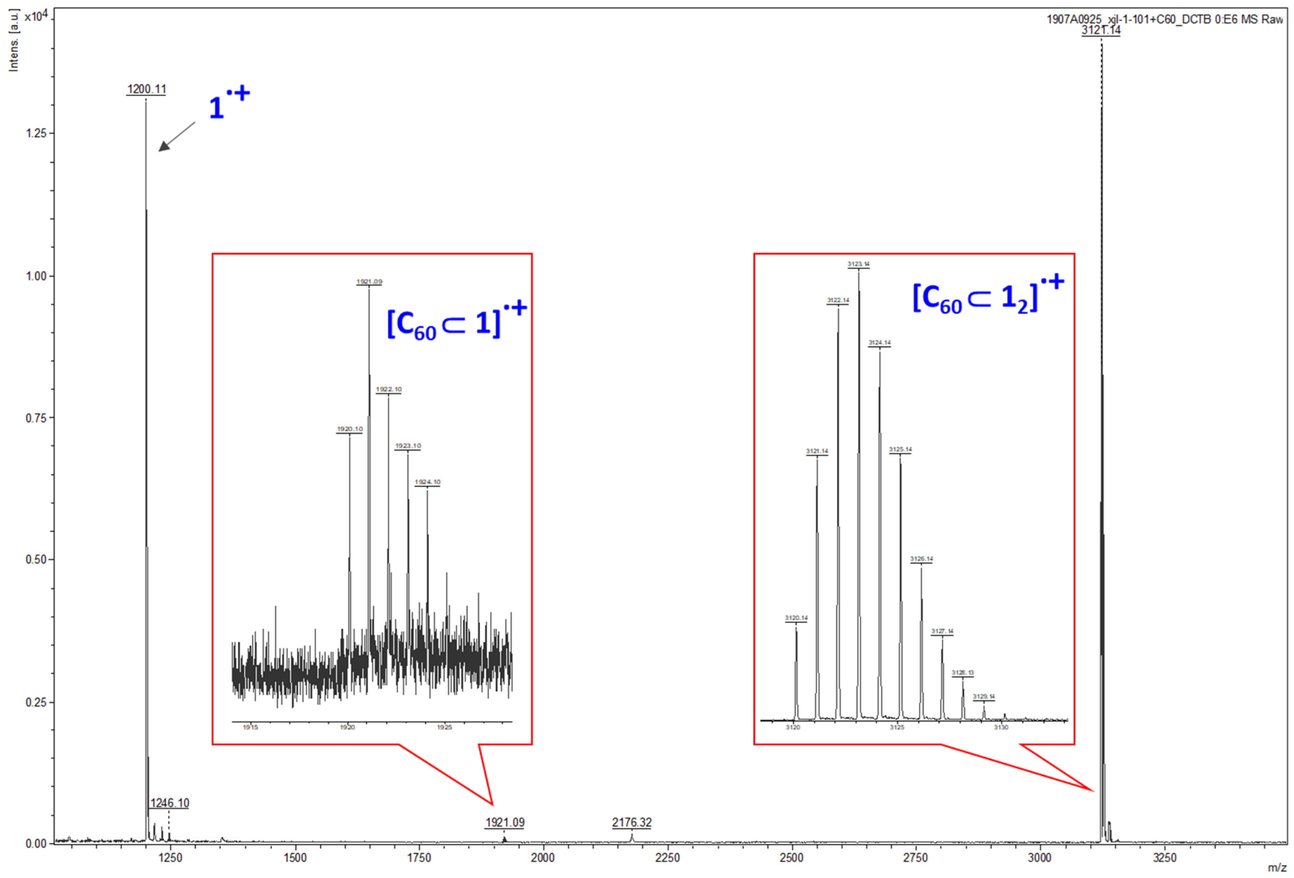

Supplementary Figure 9. MALDI-TOF-Mass spectrum of a mixed sample of **1** and **C_{60}** with a mole ratio of 2:1 in *o*-dichlorobenzene. The isotope distribution of the observed peaks for the 1:1 complex (*m/z* = 1920.10) and 2:1 complex (*m/z* = 3120.14) were amplified.

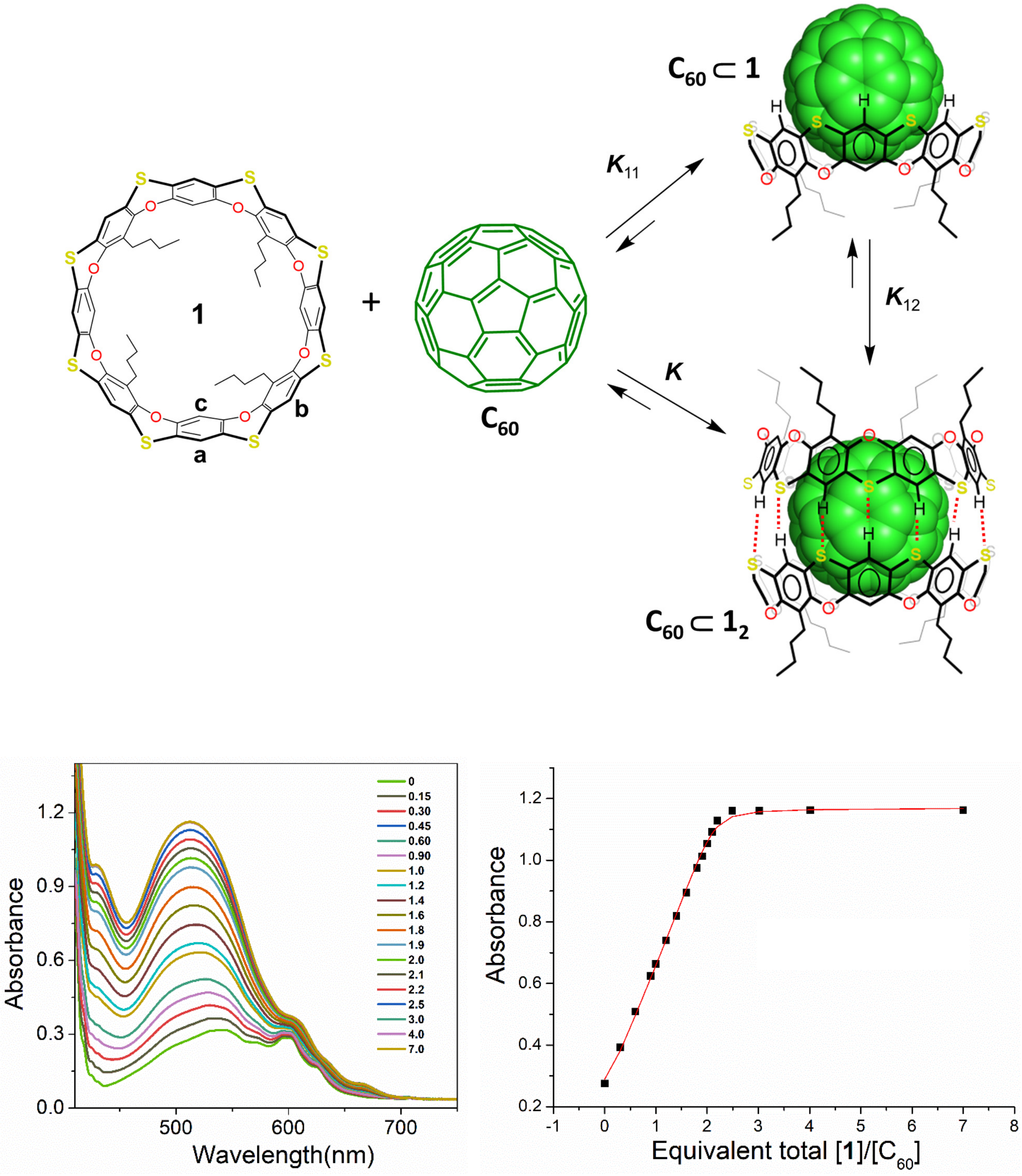

Supplementary Figure 10. UV/Vis absorption spectral changes of fullerene C_{60} (0.3 mM) in *o*-dichlorobenzene upon addition of **1** from 0 to 7.0 equiv in a 1.0 cm path cuvette while the concentration of C_{60} was maintained constant. Association constants, $K_{11} = 1.3 \times 10^4\ M^{-1}$, $K_{12} = 2.8 \times 10^5\ M^{-1}$, $K = K_{11} \cdot K_{12} = 3.6 \times 10^9\ M^{-2}$, were obtained by a global fitting analysis to a 2:1 binding model using L-BFGS-B method with Bindfit.[4, 5]

Host-guest chemistry of 2 with [2.2]paracyclophane

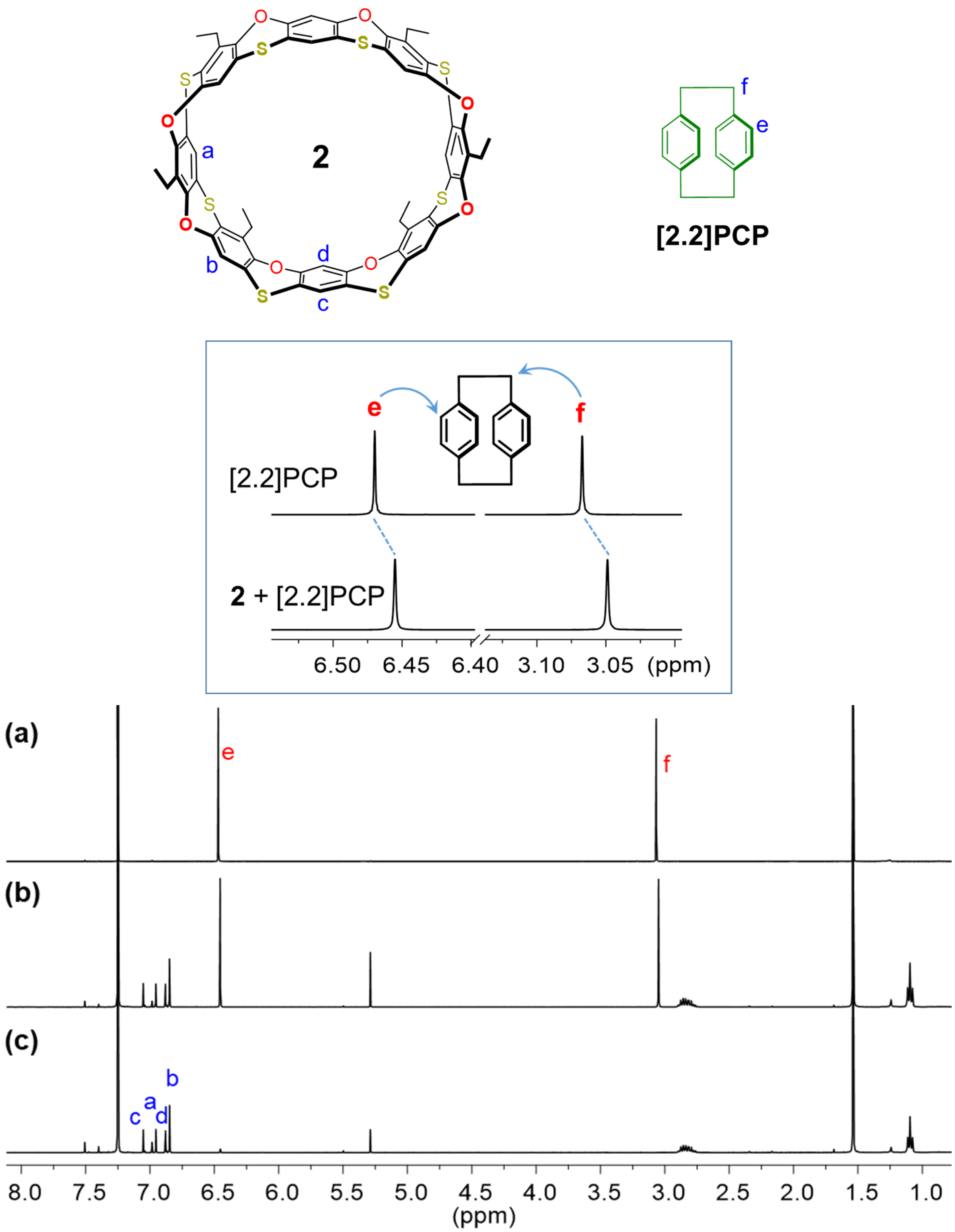

Supplementary Figure 11. ^{1}H NMR spectra (400 MHz, 298 K, $CDCl_3$) of a) 1 mM of [2.2]paracyclophane; b) an equimolar mixture of **2** and [2.2]paracyclophane; and c) 1 mM of **2**. The upfield chemical shifts of protons H_e and H_f of [2.2]paracyclophane indicating shielding effect which could be resulted from the formation of inclusion complex.

A solution of **2** in $CDCl_3$ (1.0×10^{-3} M, 5 mL) was prepared (solution **A**). An excess of guest [2.2] paracyclophane (6 equivalents and 50 equivalents relative to host **2**) in solution **A** were then prepared (solution **B**) and (solution **C**). A 0.600 mL of initial volume of solution **A** was titrated with increasing amounts of solution **B** and solution **C**. A ^{1}H NMR spectrum was recorded after each addition. All the spectra were recorded after shaking the NMR tube thoroughly, in order to allow the host-guest systems to reach the equilibrium. After referencing each spectrum, the δ values for the host **2** resonance were extracted and fit to a 1:1 binding isotherm using Bindfit.[S3, S4]

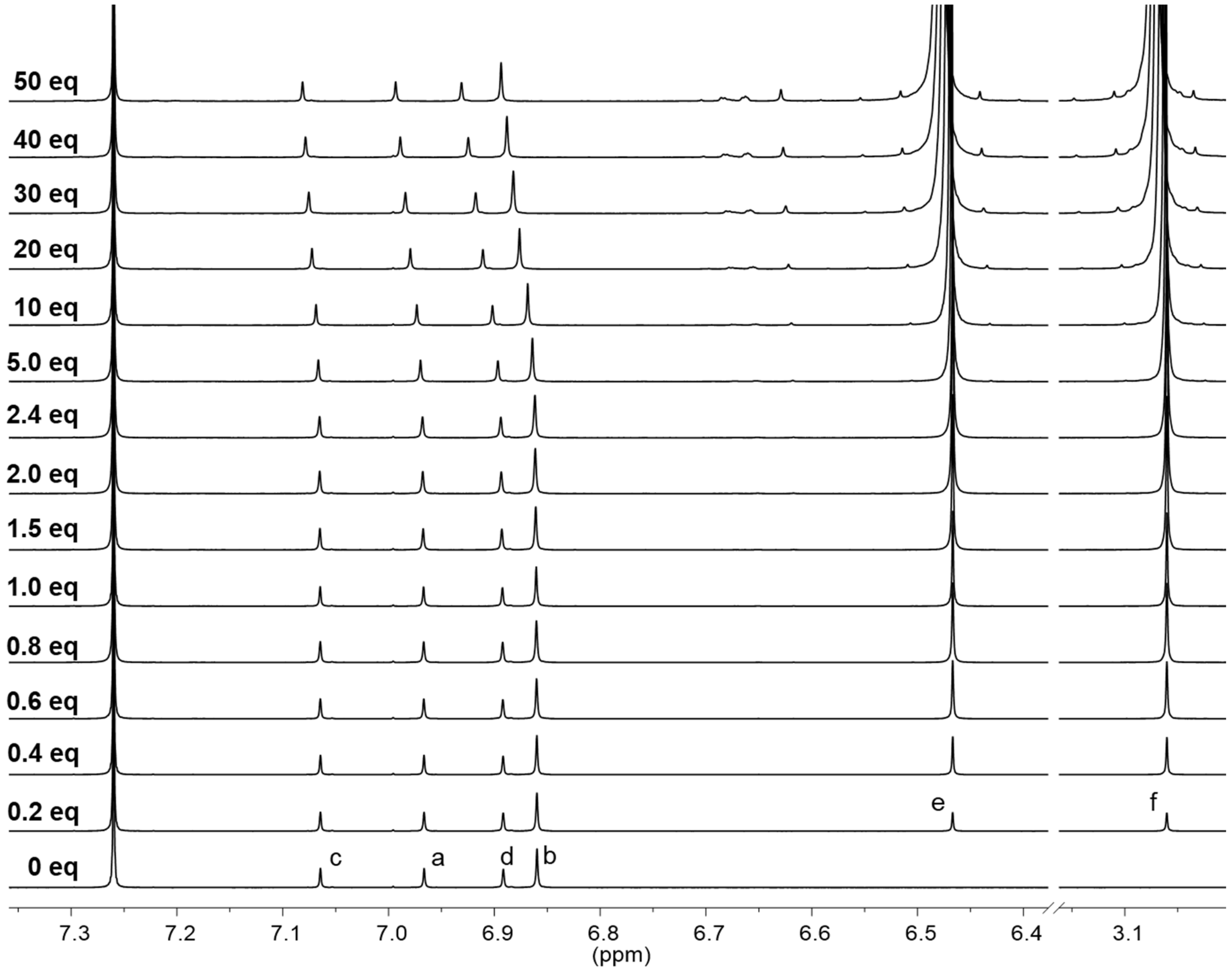

Supplementary Figure 12. ^{1}H NMR spectra (400 MHz, 298 K) of **2** (1mM) in $CDCl_3$ after adding different amount (from 0 to 50 eq.) of [2.2]paracyclophane relative to **2**.

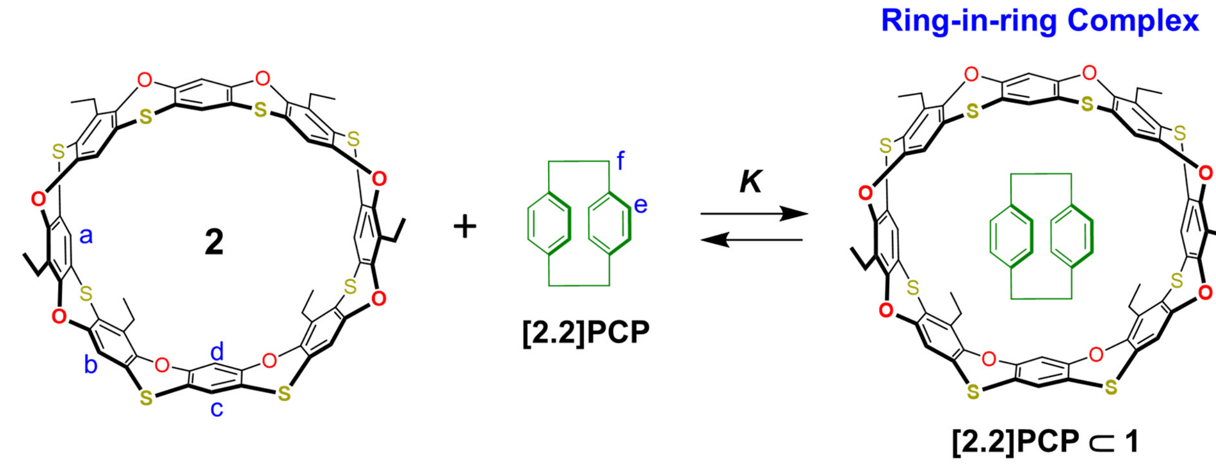

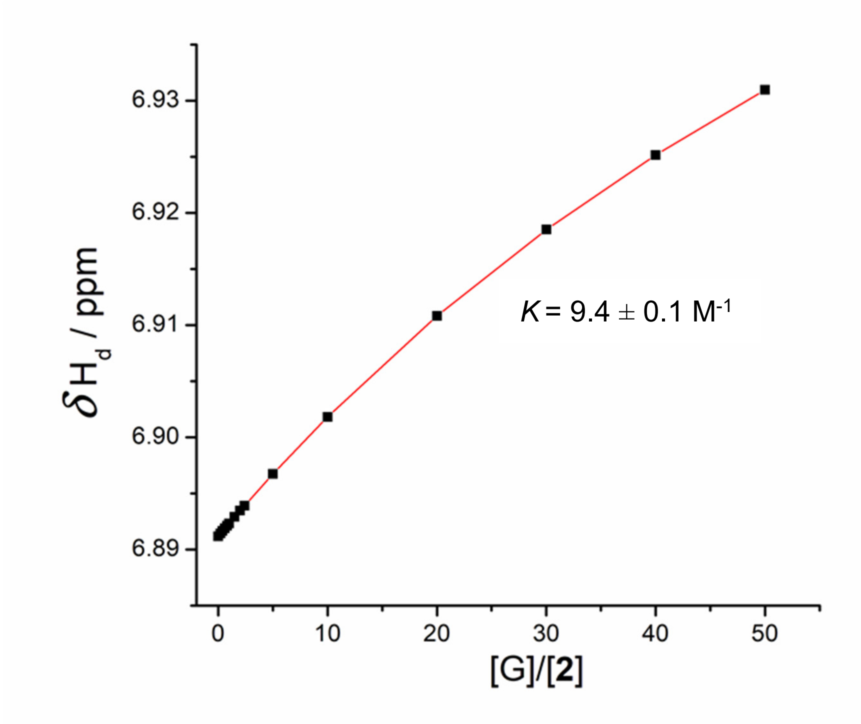

Supplementary Figure 13. Non-linear fitting curve of the resonance of proton H_d of **2** upon titrated with [2.2]paracyclophane. By using a 1:1 binding model, the binding constant was be determined using Bindfit.[S3, S4]

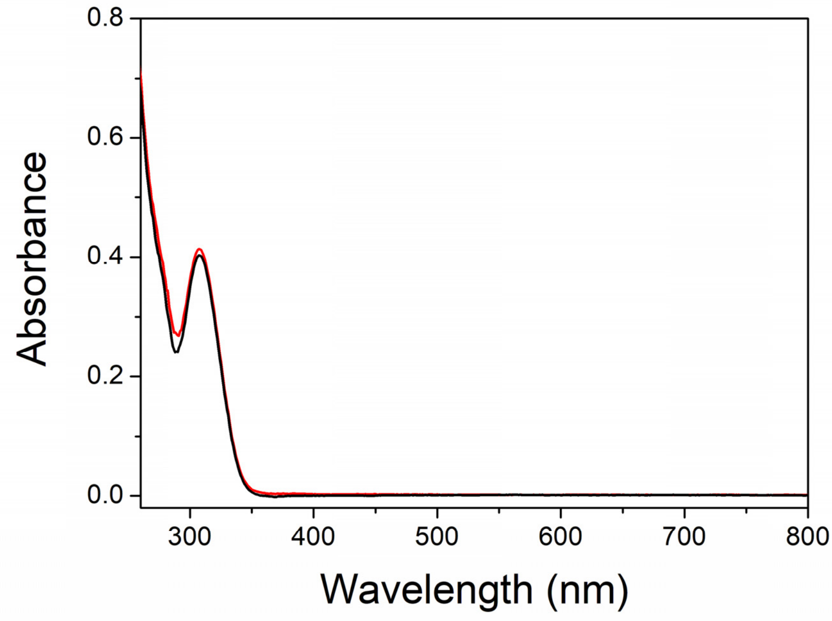

Supplementary Figure 14. UV/Vis absorption spectra of **2** (6.0×10^{-4} M, black line), and a mixed sample of **2** (6.0×10^{-4} M) with 1 equivalent of [2.2]paracyclophane (red line) in chloroform.

Details of X-ray Crystallography

Reflection data for **1**, C_{60}⊂**1**$_2$, and **2** were collected on a Rigaku SuperNova, Dual, AtlasS2 diffractometer using monochromatized Cu Kα radiation. Reflection data of [2.2]PCP⊂**2** was collected a BRUKER D8 VENTURE PHOTON II diffractometer using MoKα radiation. Crystals were frozen in paratone oil inside a cryoloop under a cold stream of N_2. Diffraction data and unit-cell parameters were consistent with assigned space groups. Lorentzian polarization corrections and empirical absorption corrections, based on redundant data at varying effective azimuthal angles, were applied to the data sets. The structures were solved using OLEX^2 crystallography software.[6,7] When practical, non-hydrogen atoms were refined anisotropically and hydrogen atoms placed in idealized positions and refined using a riding model. Figures were drawn with Diamond software. Details can be obtained from the Cambridge Crystallographic Data Centre at www.ccdc.cam.ac.uk for CCDC accession numbers 1978840 to 1978843.

Single-crystal X-ray structure of [2.2]PCP ⊂ 2. The unit cell contained two molecules of **2**. Each **2** had a disordered [2.2]PCP molecule in the cavity over two positions. The favoured position of the guest molecule [2.2]PCP in each complex is shown below in ball-and-stick representation.

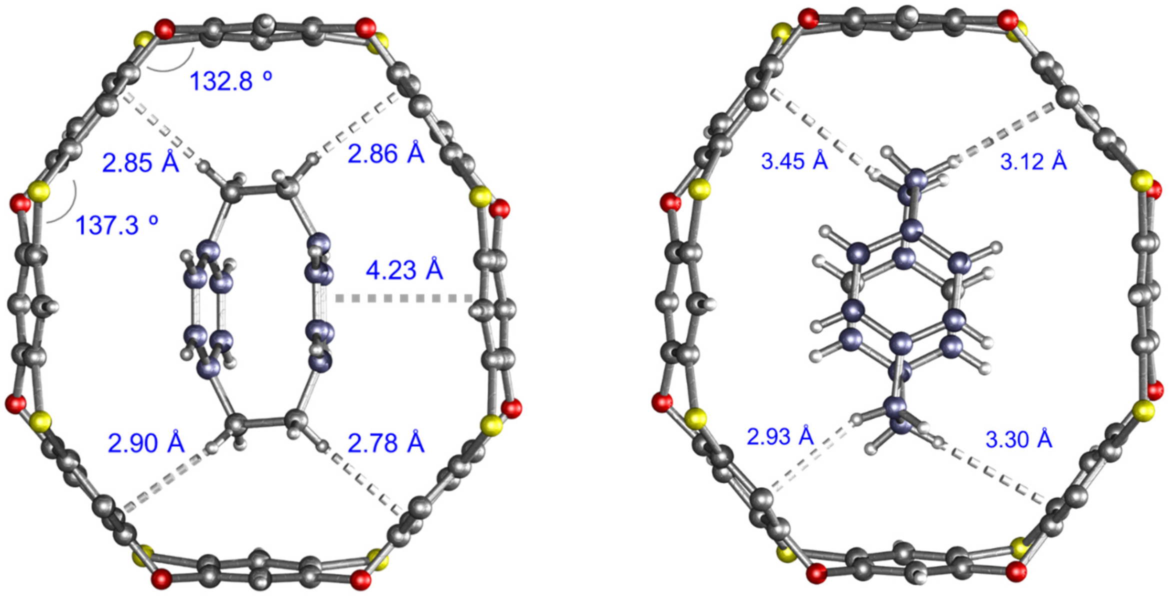

Supplementary Figure 15. Single-crystal X-ray structure of [2.2]PCP ⊂ **2**. The C-H···π interactions are highlighted in white dash lines. Color code: S = gold, O = red, Cl = green, C = black or gray slate-blue, H = white.

Supplementary Table 1. Crystal Data, Solution and Refinement Parameters.

	$\mathbf{1}\cdot(C_6H_5NO_2)_{2.5}$	$[C_{60}\subset\mathbf{1}_2]\cdot(C_6H_4Cl_2)\cdot(C_6H_{14})_{0.5}$	$\mathbf{2}\cdot(C_6H_4Cl_2)_3\cdot(C_6H_{14})$	[2.2]PCP $\subset$ **2**
**CCDC number**	1978840	1978841	1978842	1978843
**formula**	$C_{79}H_{60.5}N_{2.5}O_{13}S_8$	$C_{197}H_{107}Cl_2O_{16}S_{16}$	$C_{84}H_{66}Cl_6O_8S_8$	$C_{76}H_{56}O_8S_8$
**formula weight**	1509.28	3313.69	1672.54	1353.69
**crystal system**	Triclinic	Monoclinic	Monoclinic	Triclinic
**space group**	*P*-1	*C*2/c	*P*21/n	*P*-1
**T (K)**	150(2)	150(2)	150(2)	190(2)
**a (Å)**	14.9042(3)	25.2945(4)	12.7817(2)	15.9183(10)
**b (Å)**	16.4925(3)	20.5468(3)	31.7627(5)	17.8162(9)
**c (Å)**	16.7551(4)	29.9744(5)	19.0999(3)	25.0737(12)
**α (º)**	114.186(2)	90.00	90	89.846(3)
**β (º)**	107.393(2)	101.052(2)	90.9630(10)	89.538(3)
**γ (º)**	94.895(2)	90.00	90	64.313(2)
**V ($Å^3$)**	3481.58(16)	15289.4(4)	7753.1(2)	6408.0(6)
**Z**	2	4	4	4
ρ, **g cm^{-3}**	1.440	1.440	1.433	1.403
μ, **mm^{-1}**	2.946	2.999	4.500	0.339
**reflections used**	13716	15007	14645	22994
**variables**	1190	1264	1220	1868
**restraints**	1120	1149	1133	401
**R_1 [$I > 2\sigma(I)$]**[a]	0.0487	0.1092	0.0766	0.1349
**R_1 (all data)**	0.0518	0.1183	0.0837	0.2082
**R_2w[$I > 2\sigma(I)$]**[b]	0.1341	0.2369	0.2007	0.2757
**R_2w (all data)**	0.1374	0.2416	0.2087	0.3173
**GoF on F^2**	1.026	1.057	1.061	1.044

[a] $R_1 = \Sigma ||F_o| - |F_c|| / \Sigma |F_o|$; [b] $R_2w = [\Sigma[w(F_o^2 - F_c^2)^2] / \Sigma[w(F_o^2)^2]]^{1/2}$, where $w = q[\sigma^2(F_o^2) + (aP)^2 + bP]^{-1}$

NMR spectra of compounds.

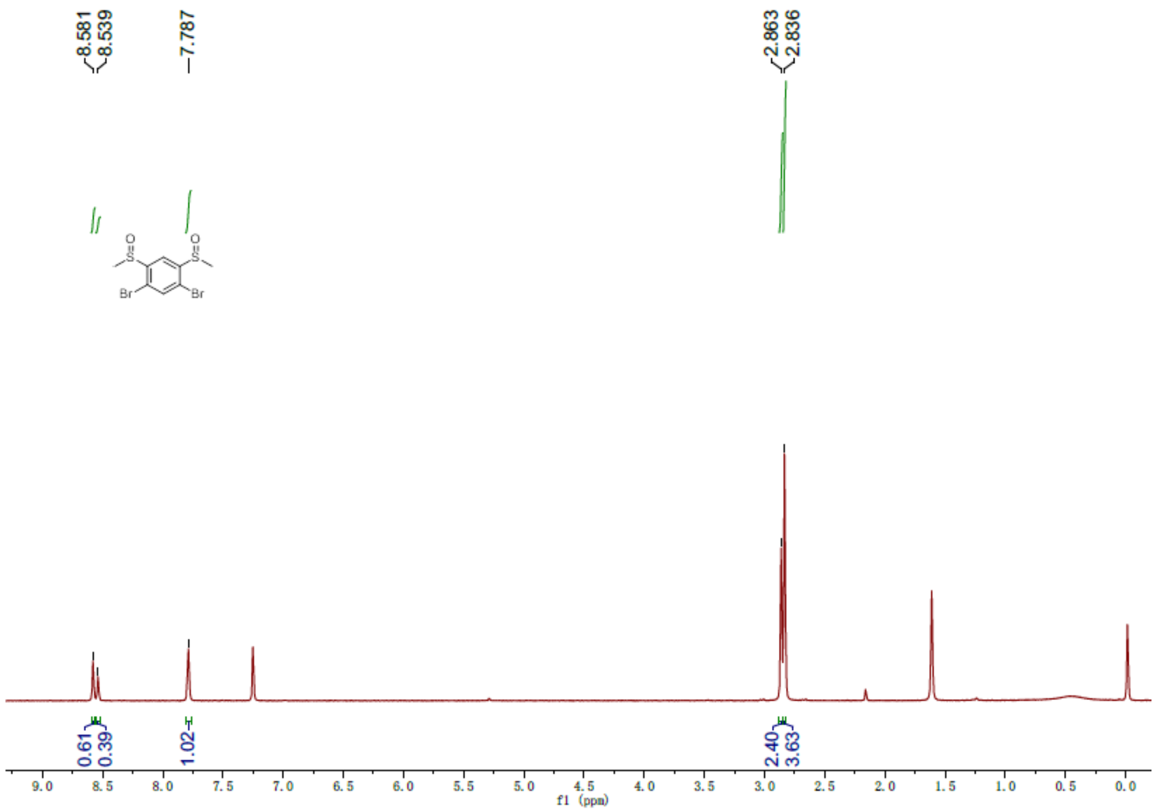

Supplementary Figure 16. ^{1}H NMR (400 MHz, $CDCl_3$, 298 K) spectrum of **C**.

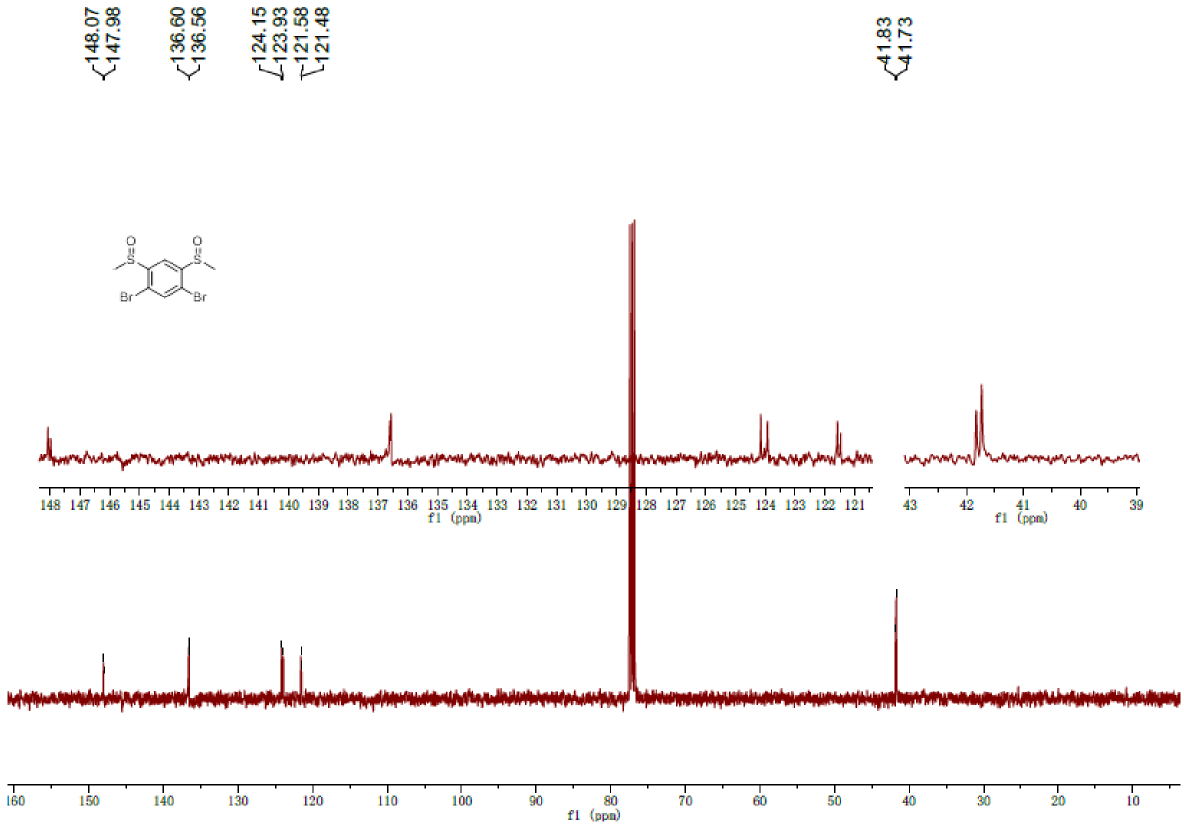

Supplementary Figure 17. ^{13}C NMR (100 MHz, $CDCl_3$, 298 K) spectrum of **C**.

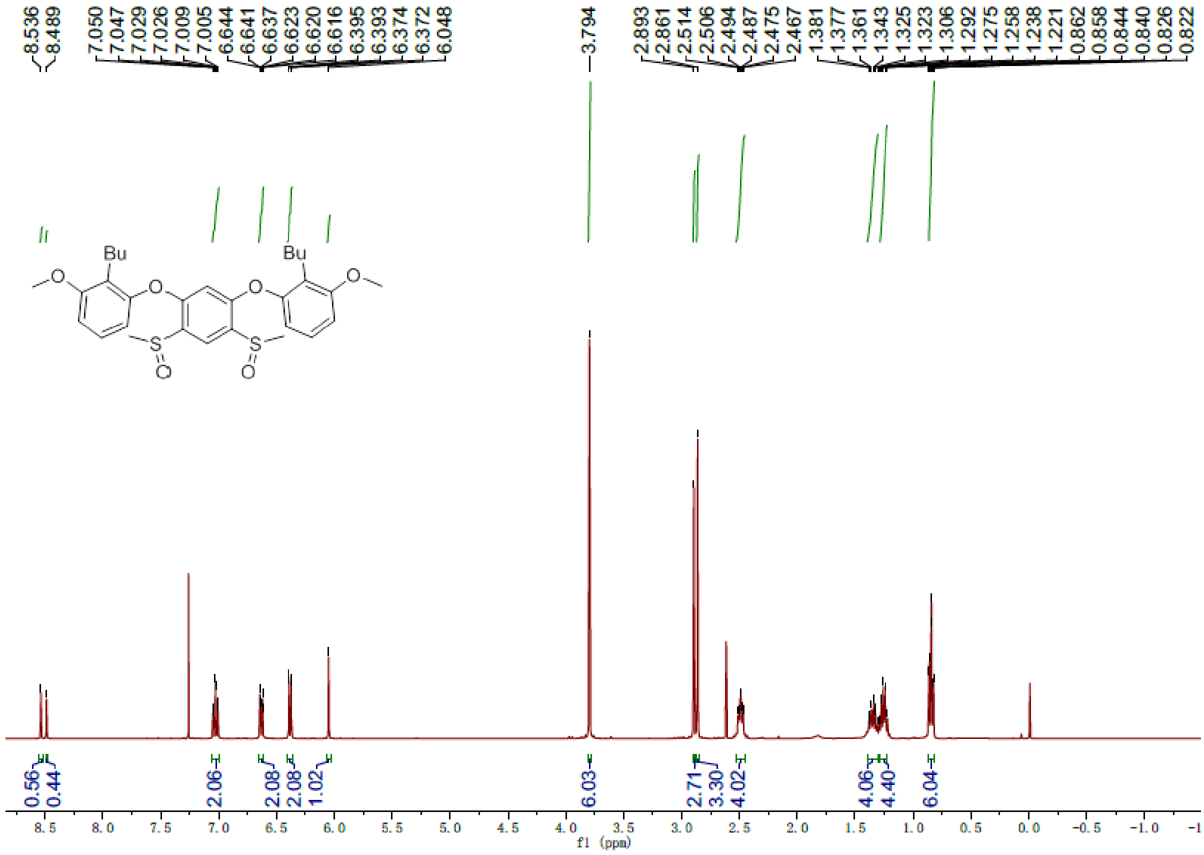

Supplementary Figure 18. ^{1}H NMR (400 MHz, $CDCl_3$, 298 K) spectrum of **4**.

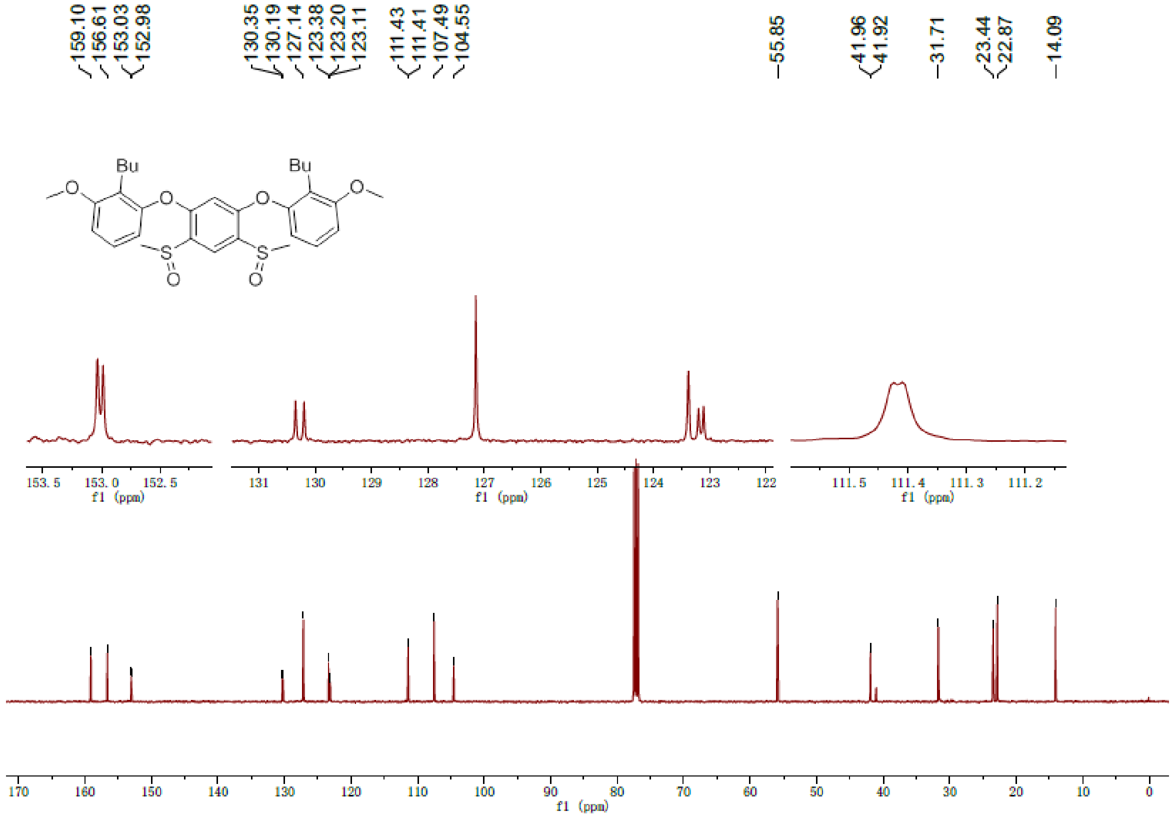

Supplementary Figure 19. ^{13}C NMR (100 MHz, $CDCl_3$, 298 K) spectrum of **4**.

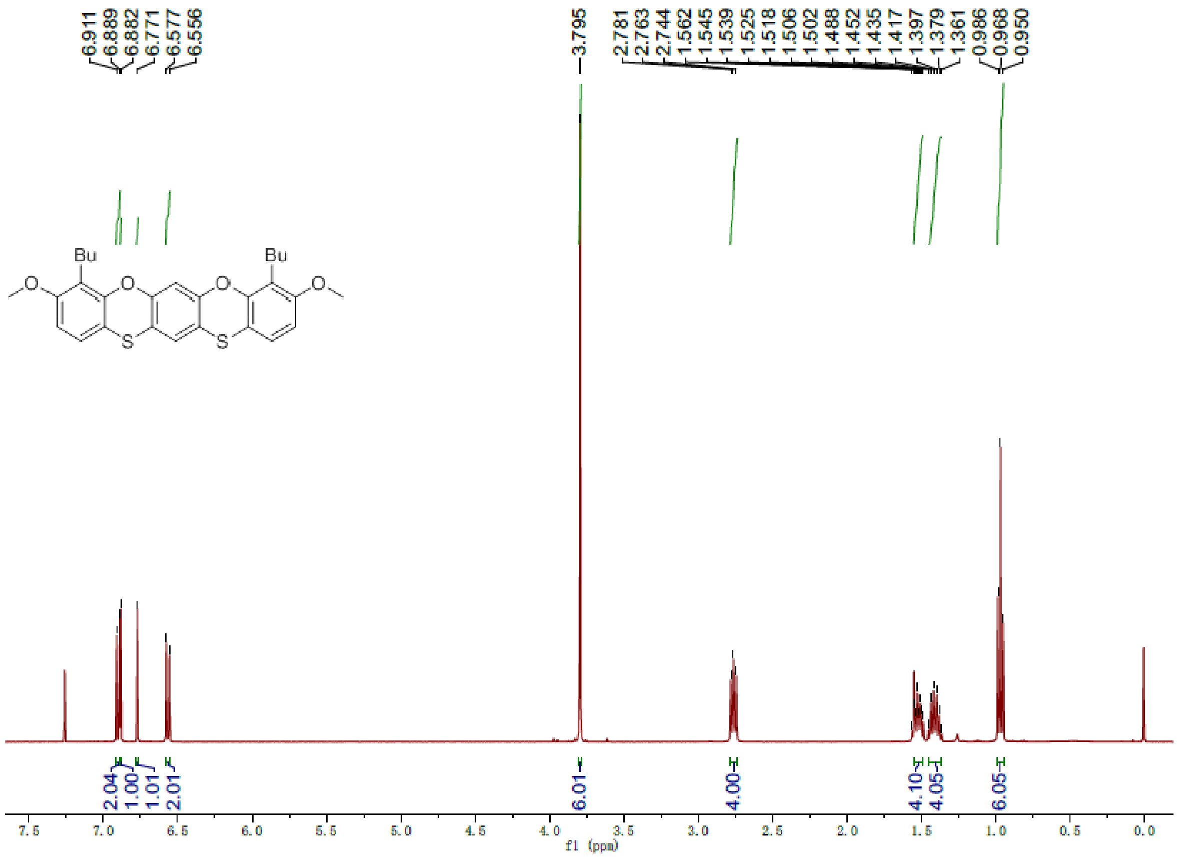

Supplementary Figure 20. ^{1}H NMR (400 MHz, $CDCl_3$, 298 K) spectrum of **5**.

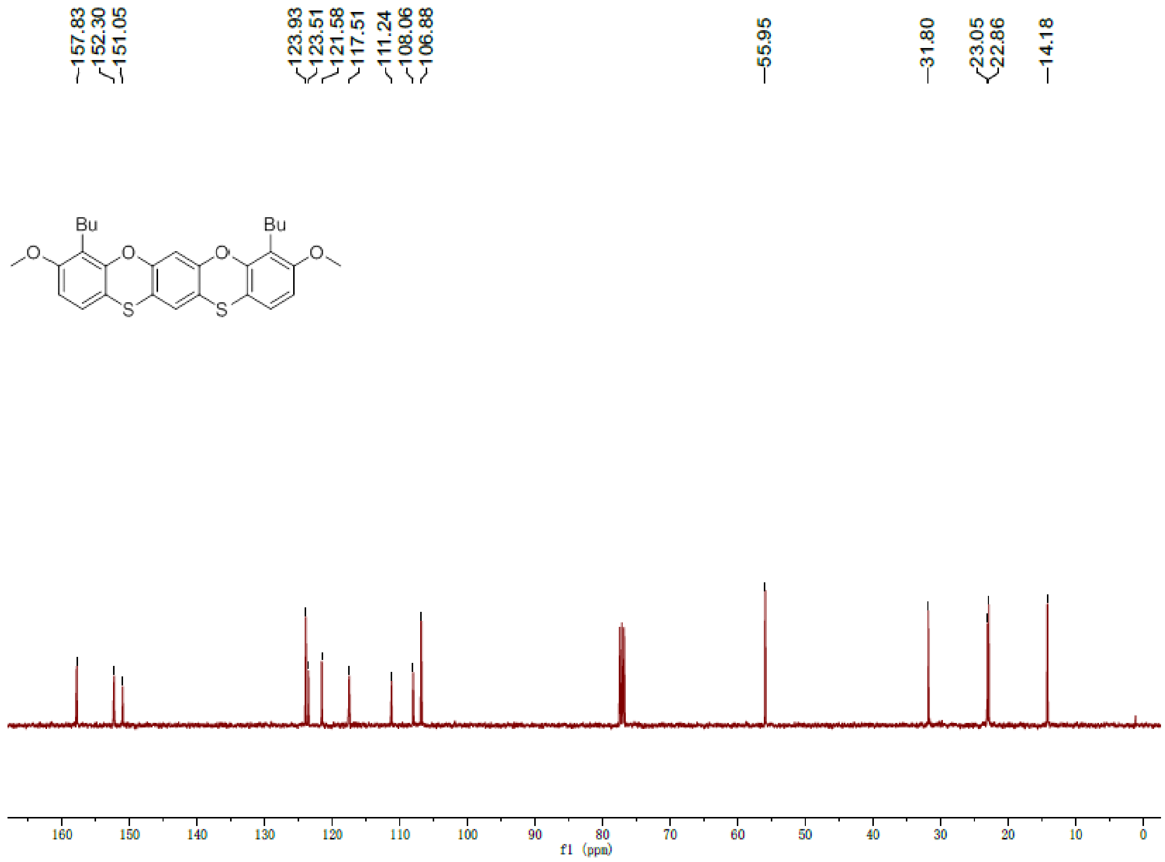

Supplementary Figure 21. ^{13}C NMR (100 MHz, $CDCl_3$, 298 K) spectrum of **5**.

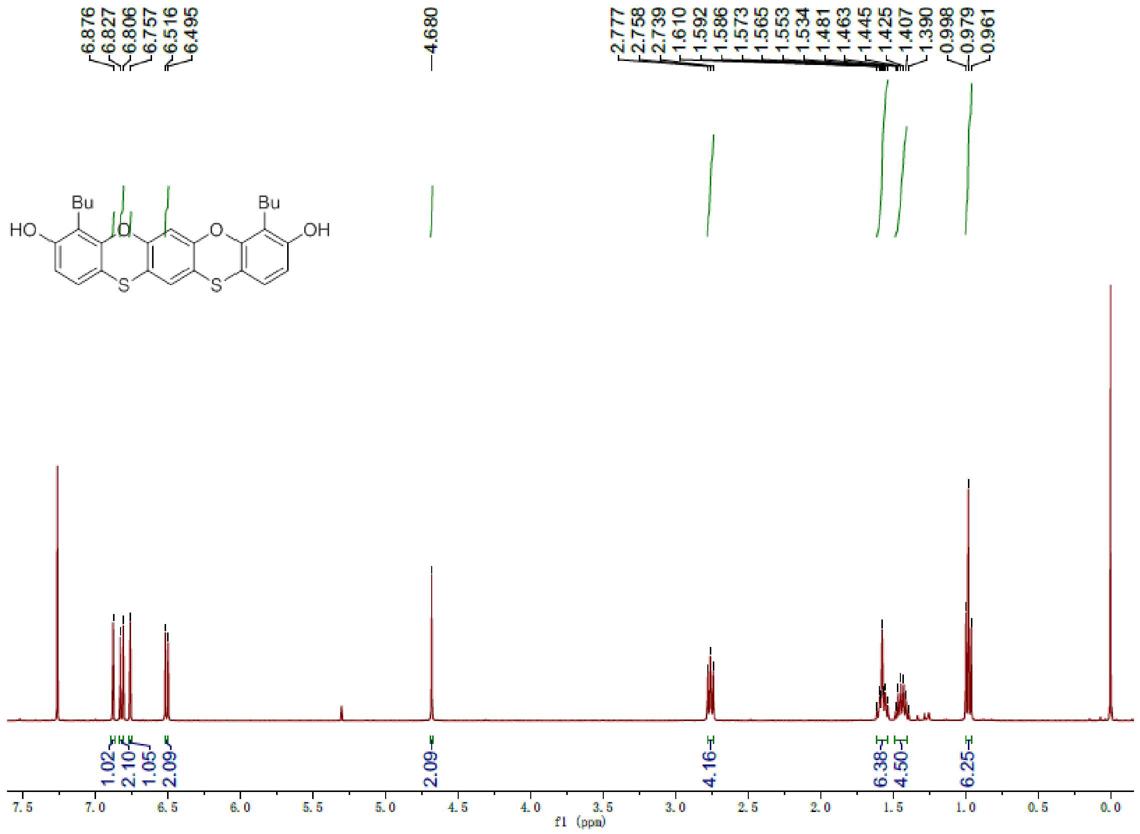

Supplementary Figure 22. ^{1}H NMR (400 MHz, $CDCl_3$, 298 K) spectrum of **A**.

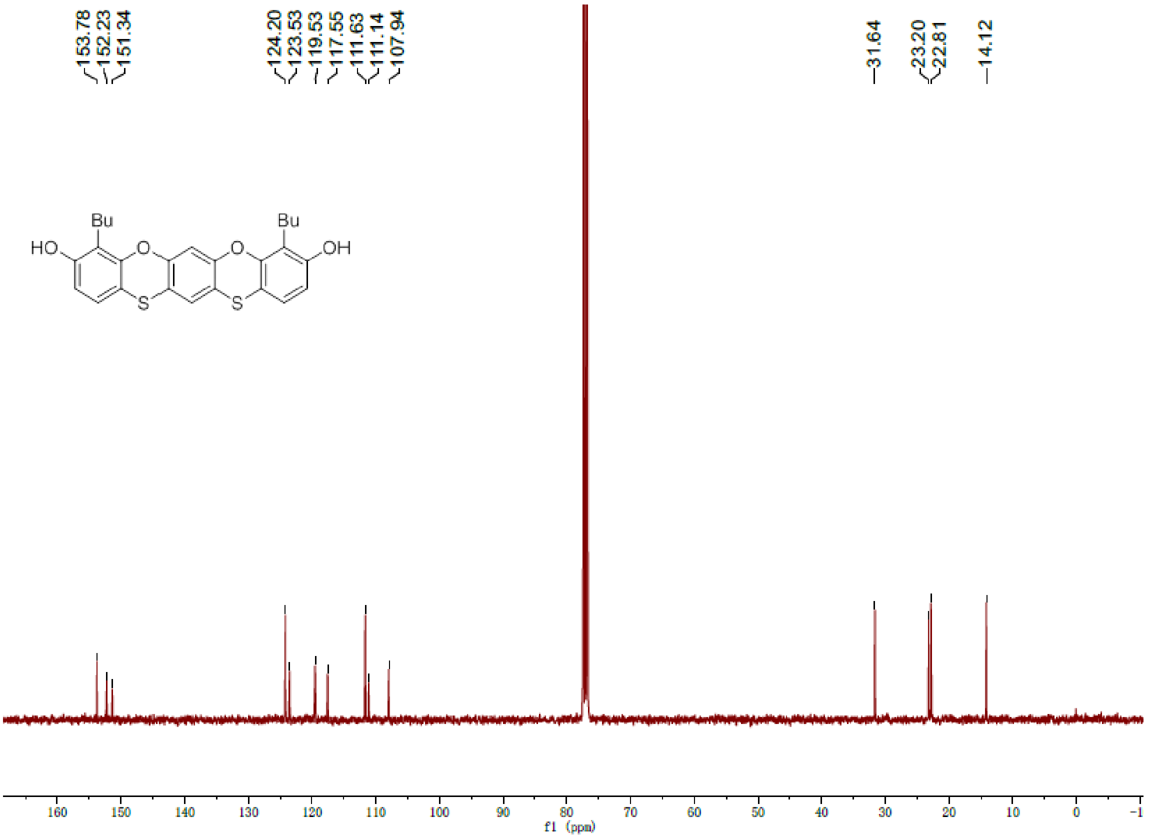

Supplementary Figure 23. ^{13}C NMR (100 MHz, $CDCl_3$, 298 K) spectrum of **A**.

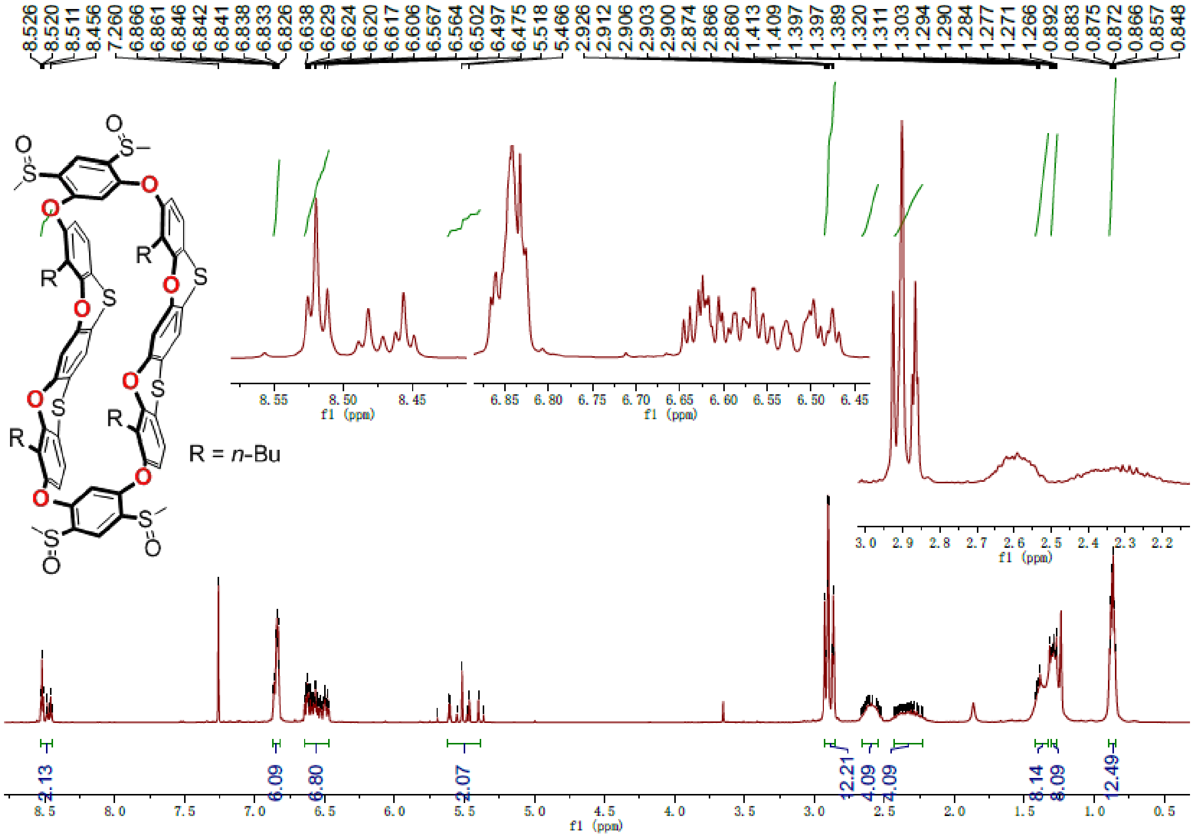

Supplementary Figure 24. ¹H NMR (400 MHz, $CDCl_3$, 298 K) spectrum of **1′**.

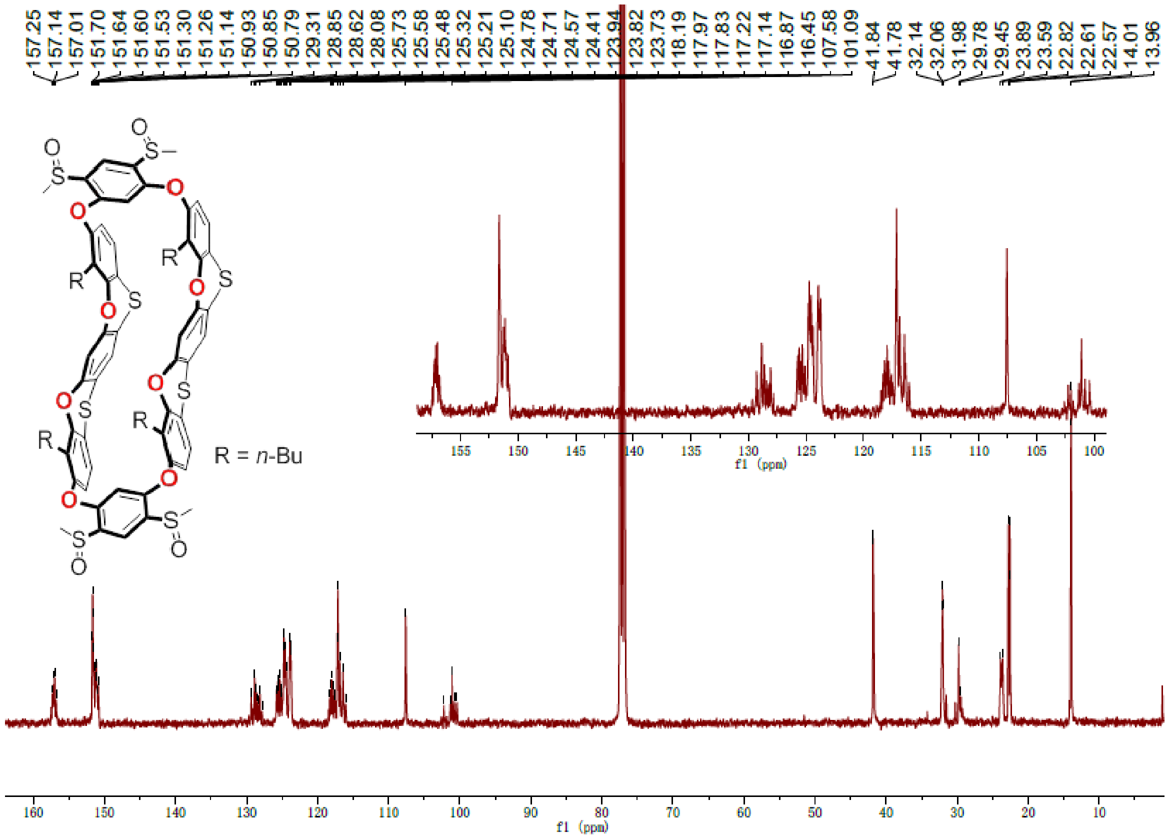

Supplementary Figure 25. ¹³C NMR (100 MHz, $CDCl_3$, 298 K) spectrum of **6′**.

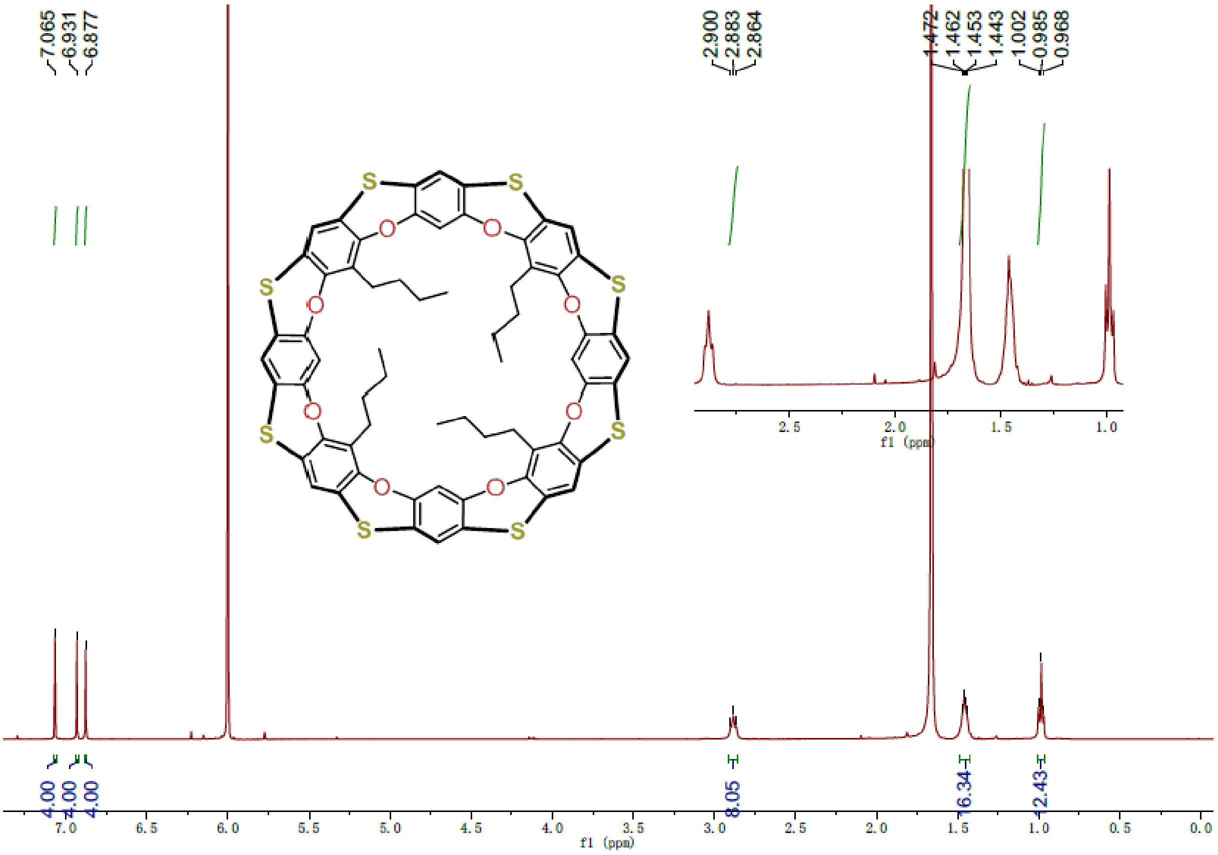

Supplementary Figure 26. ^{1}H NMR Spectrum (400 MHz, TCE-d_2, 298 K) of **1**.

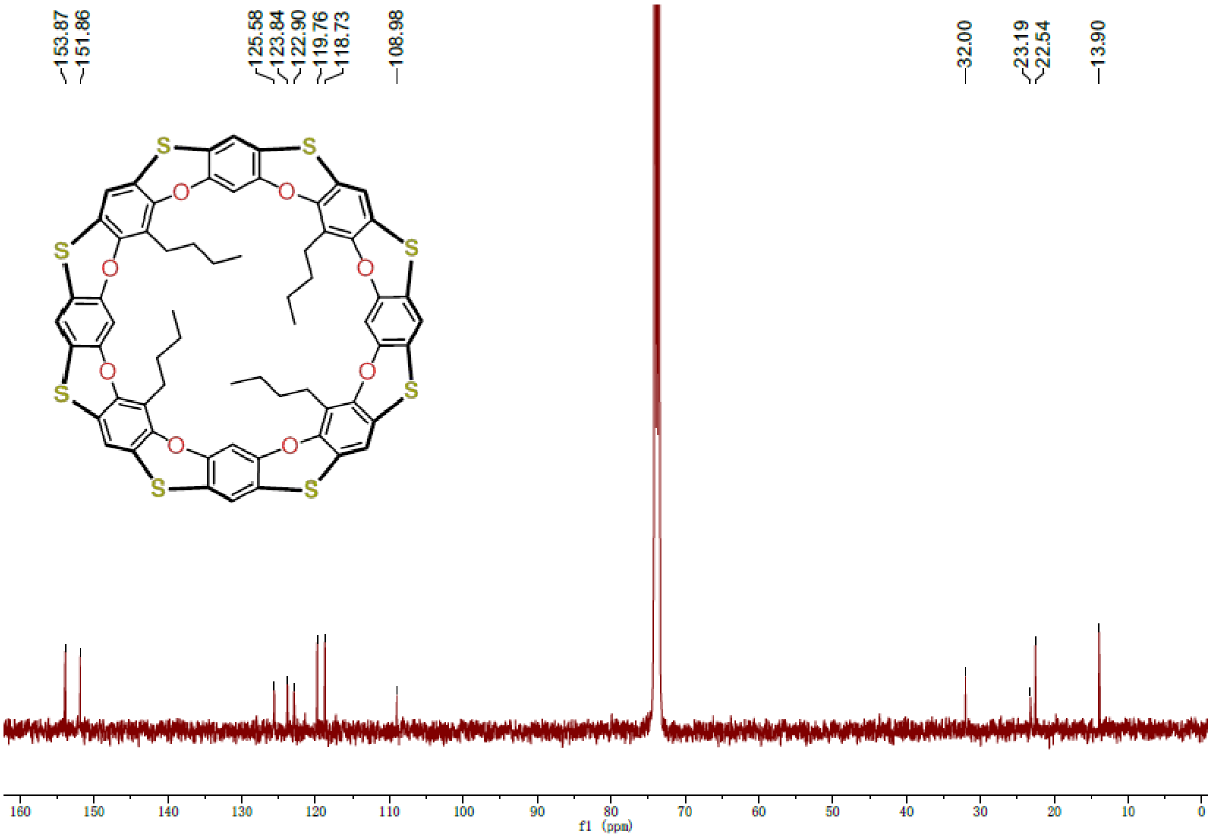

Supplementary Figure 27. ^{13}C NMR Spectrum (100 MHz, TCE-d_2, 298 K) of **1**.

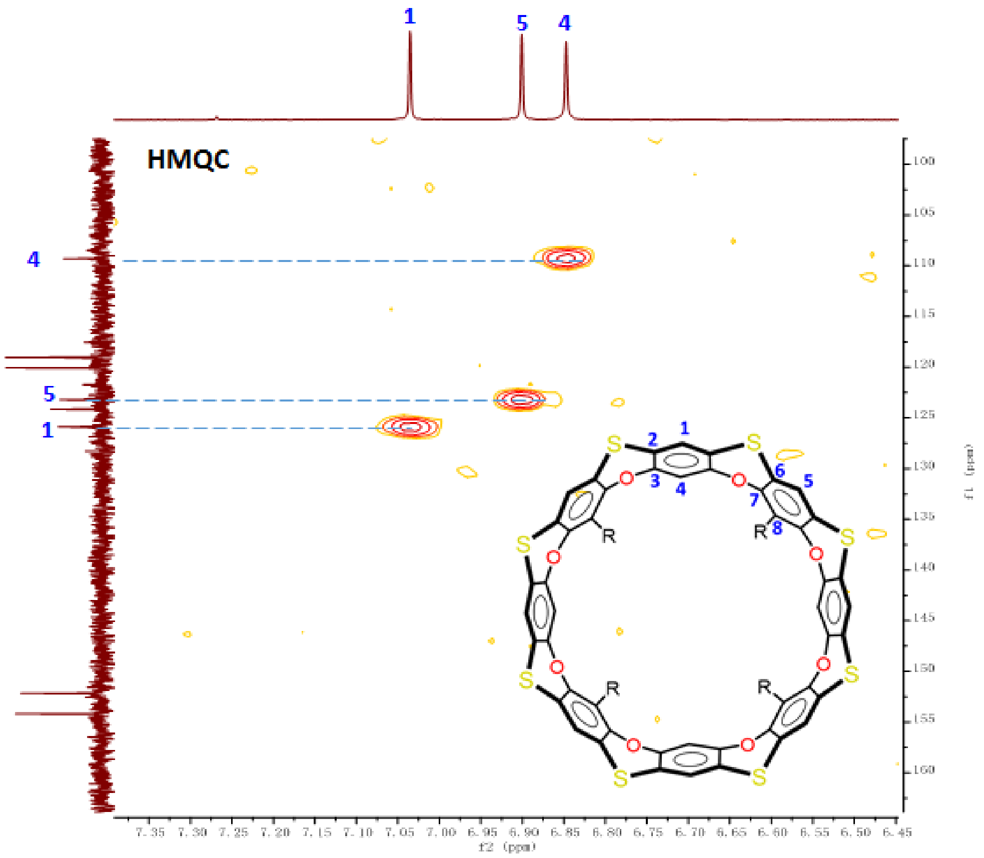

Supplementary Figure 28. ^{1}H-^{13}C HMQC NMR Spectrum (TCE-d_2, 298 K) of **1**.

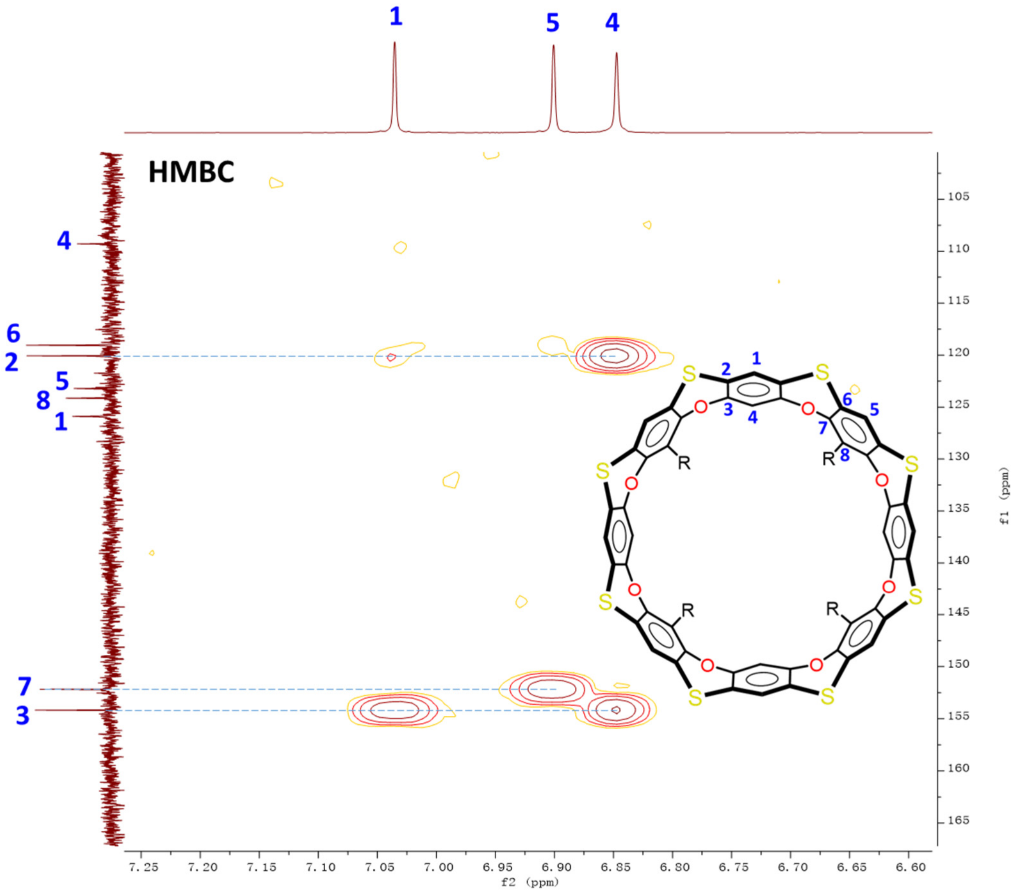

Supplementary Figure 29. ^{1}H-^{13}C HMBC NMR Spectrum (TCE-*d*2, 298 K) of **1**.

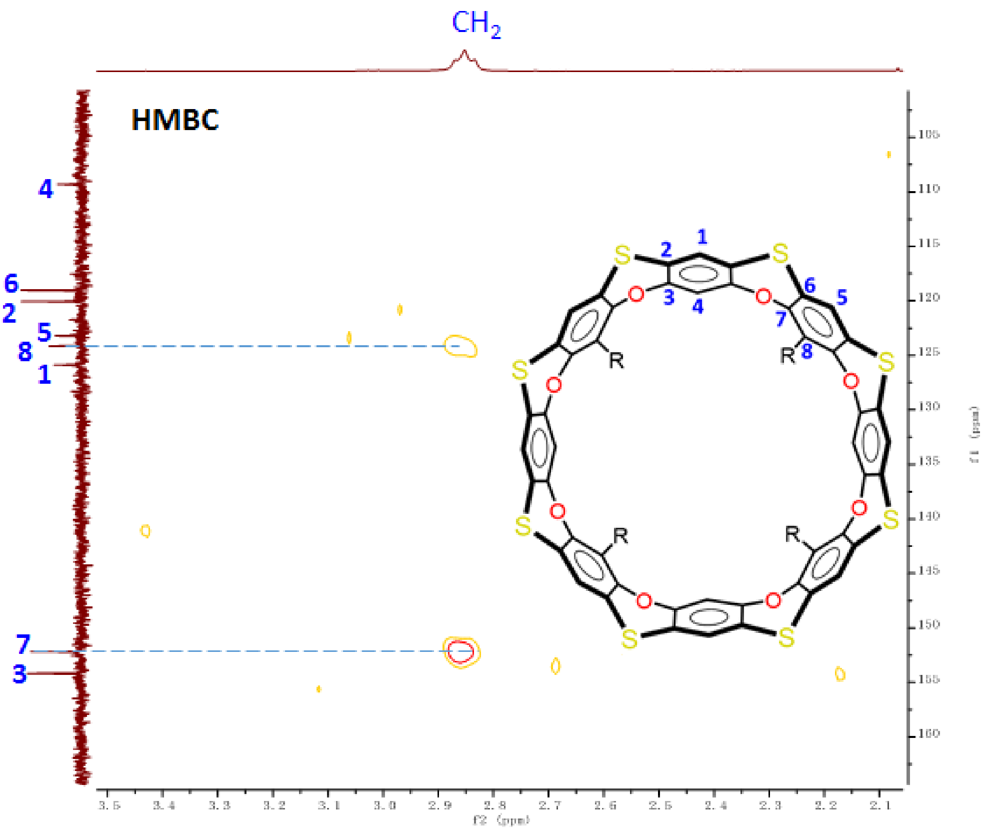

Supplementary Figure 30. ^{1}H-^{13}C HMBC NMR Spectrum (TCE-*d*2, 298 K) of **1**.

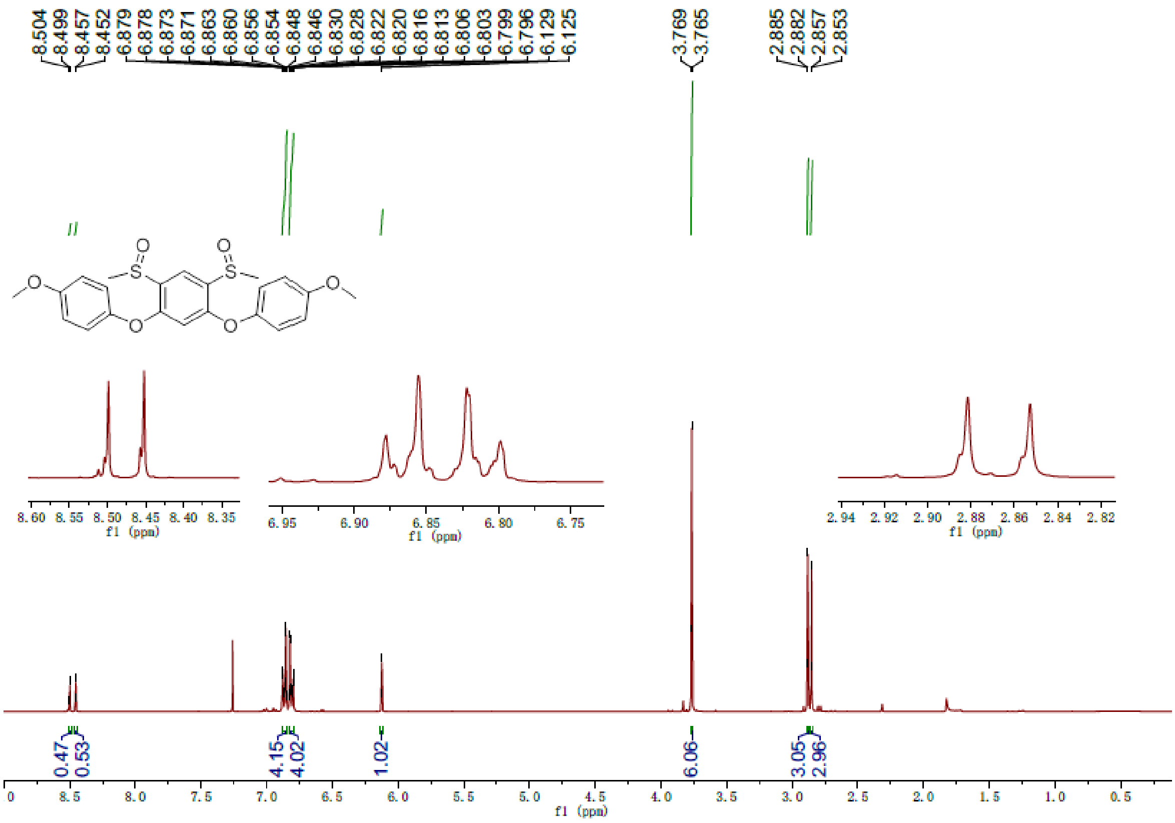

Supplementary Figure 31. ^{1}H NMR Spectrum (400 MHz, $CDCl_3$, 298 K) of **6**.

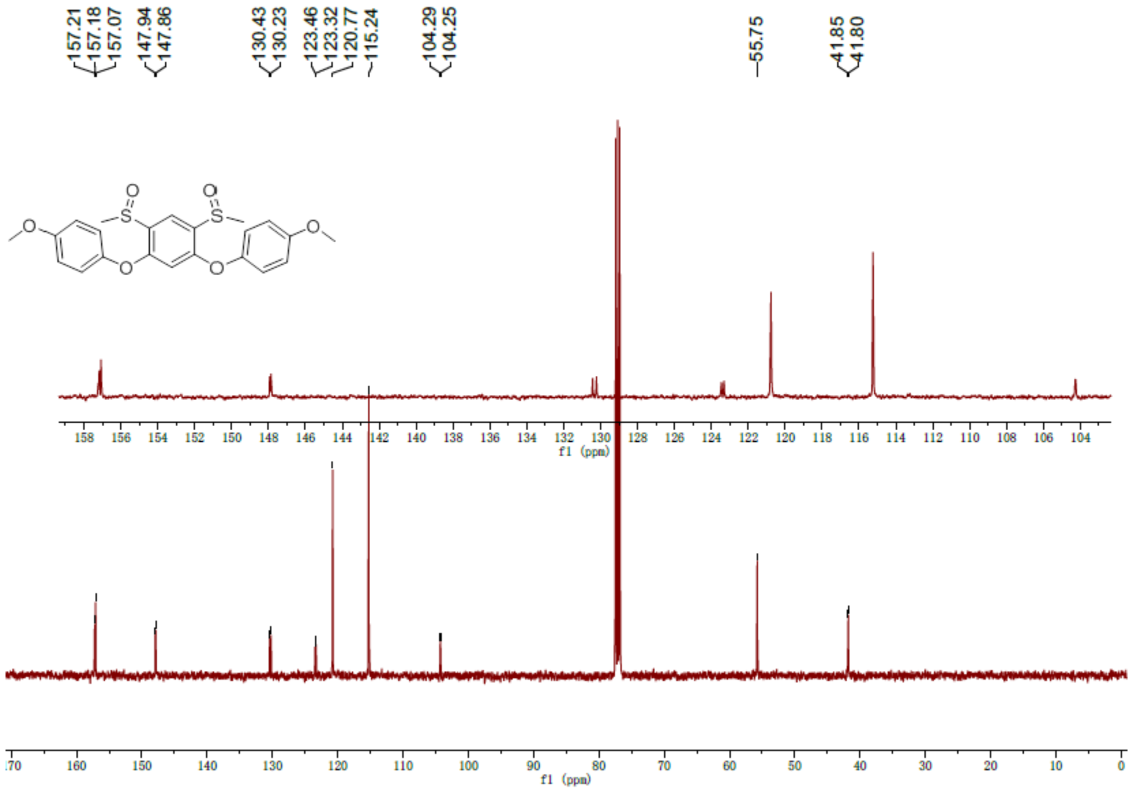

Supplementary Figure 32. ^{13}C NMR Spectrum (100 MHz, $CDCl_3$, 298 K) of **6**.

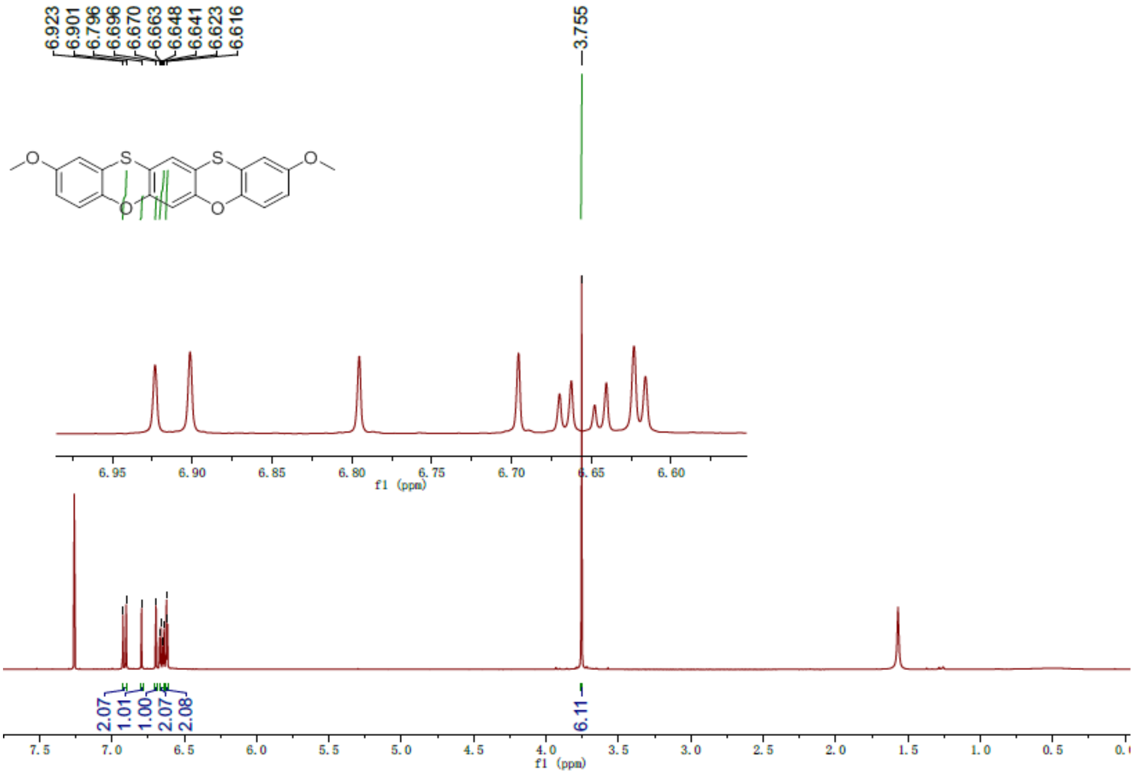

Supplementary Figure 33. ^{1}H NMR Spectrum (400 MHz, $CDCl_3$, 298 K) of **7**.

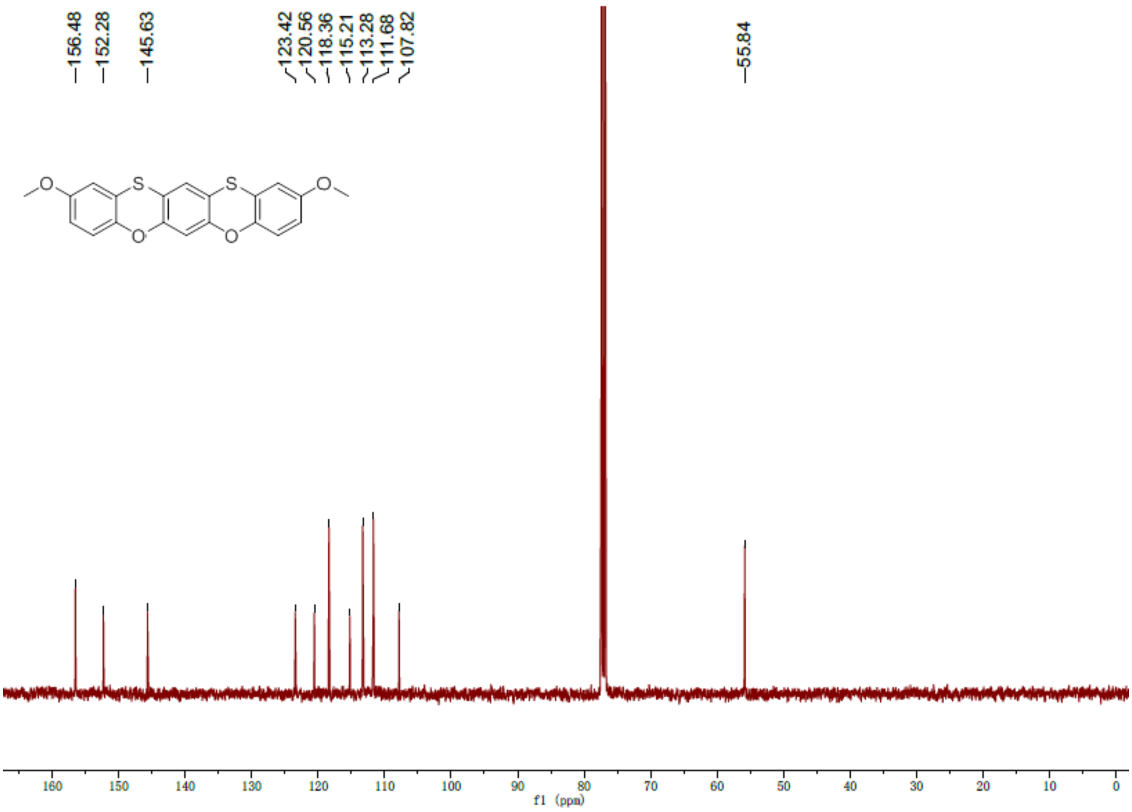

Supplementary Figure 34. ^{13}C NMR Spectrum (100 MHz, $CDCl_3$, 298 K) of **7**.

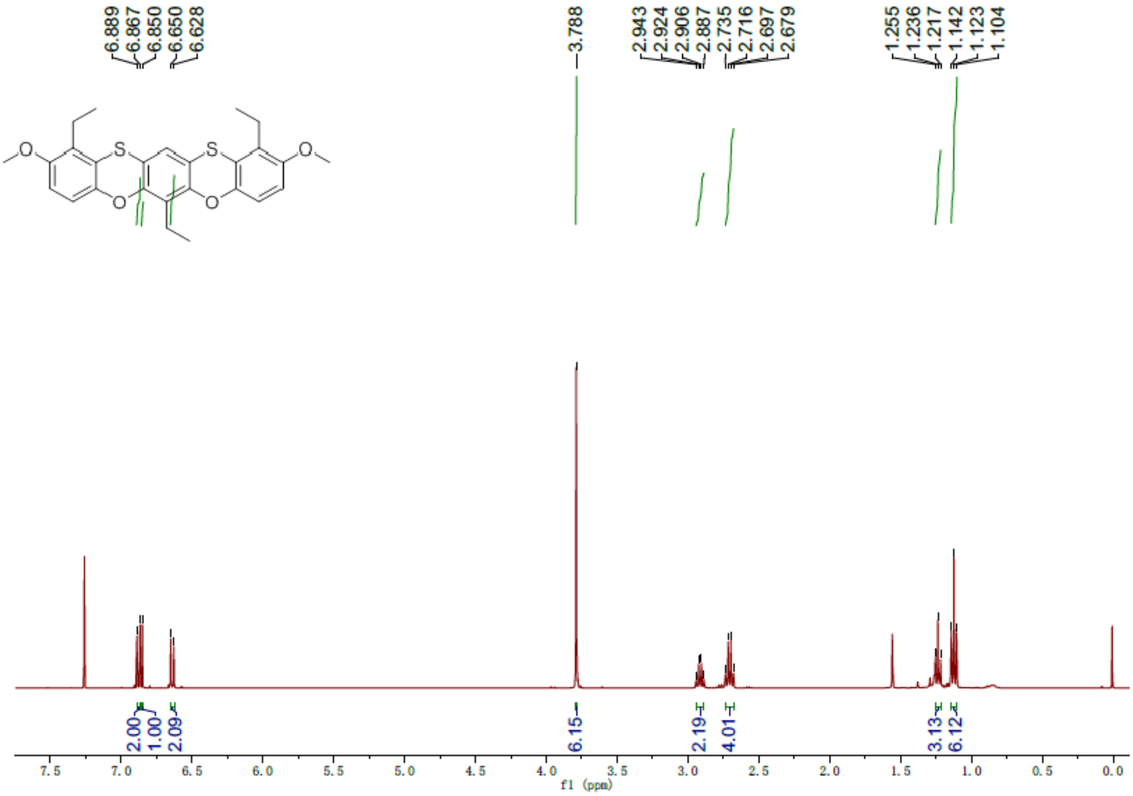

Supplementary Figure 35. ^{1}H NMR Spectrum (400 MHz, $CDCl_3$, 298 K) of **8**.

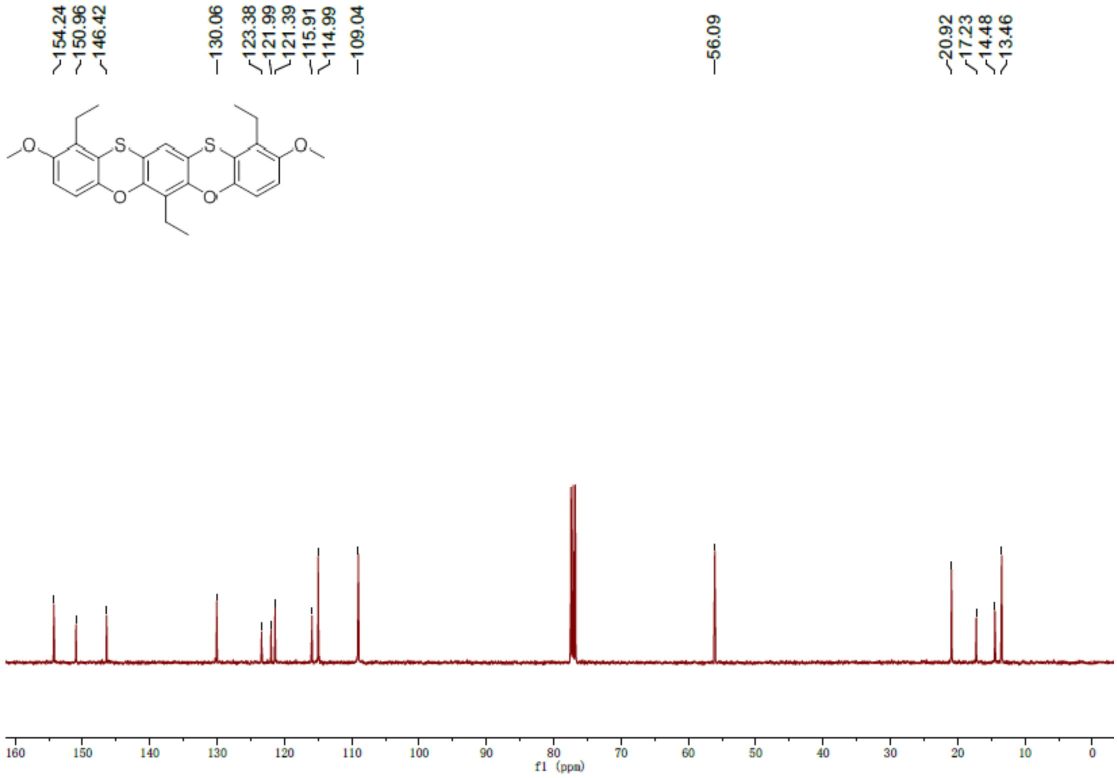

Supplementary Figure 36. ^{13}C NMR Spectrum (100 MHz, $CDCl_3$, 298 K) of **8**

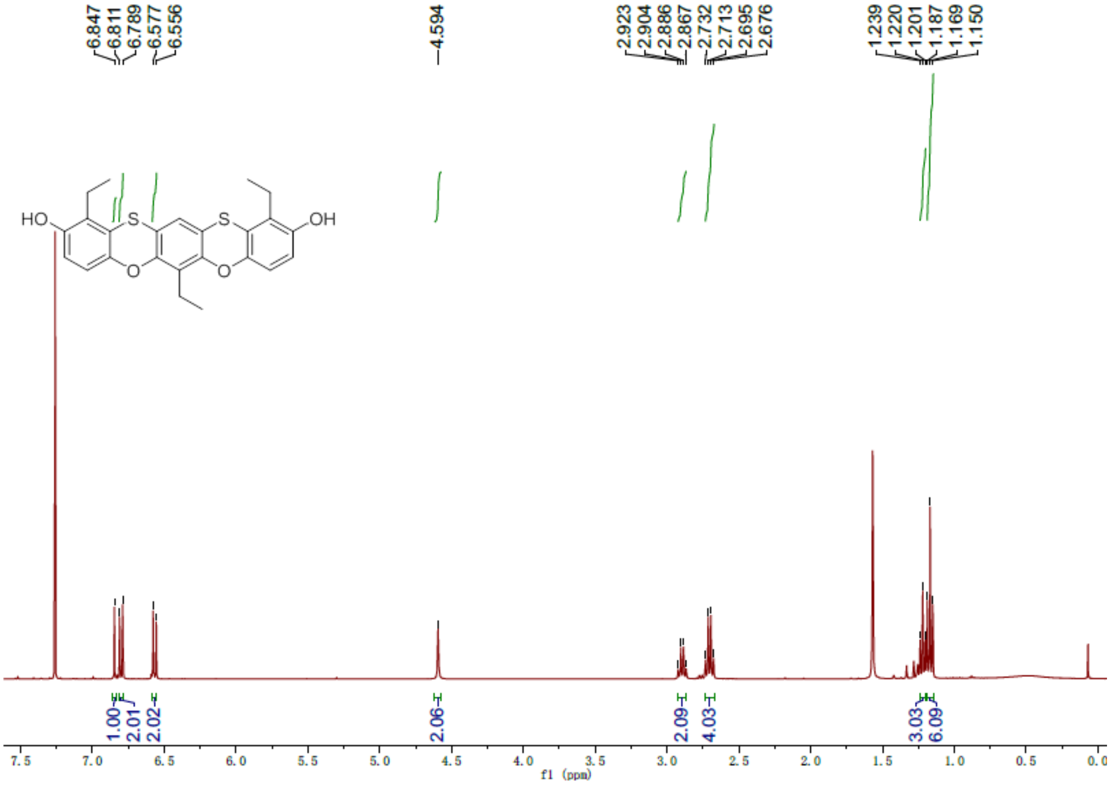

Supplementary Figure 37. ^{1}H NMR Spectrum (400 MHz, $CDCl_3$, 298 K) of **B**.

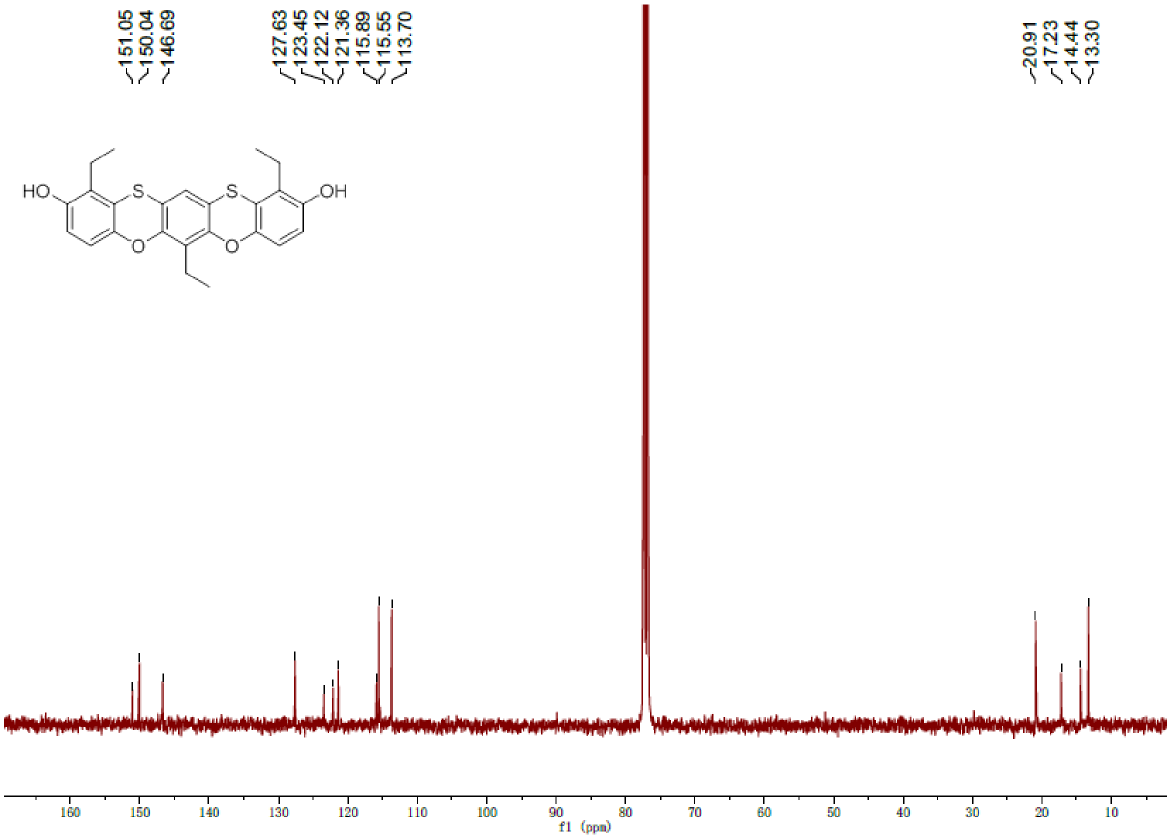

Supplementary Figure 38. ^{13}C NMR Spectrum (100 MHz, $CDCl_3$, 298 K) of **B**

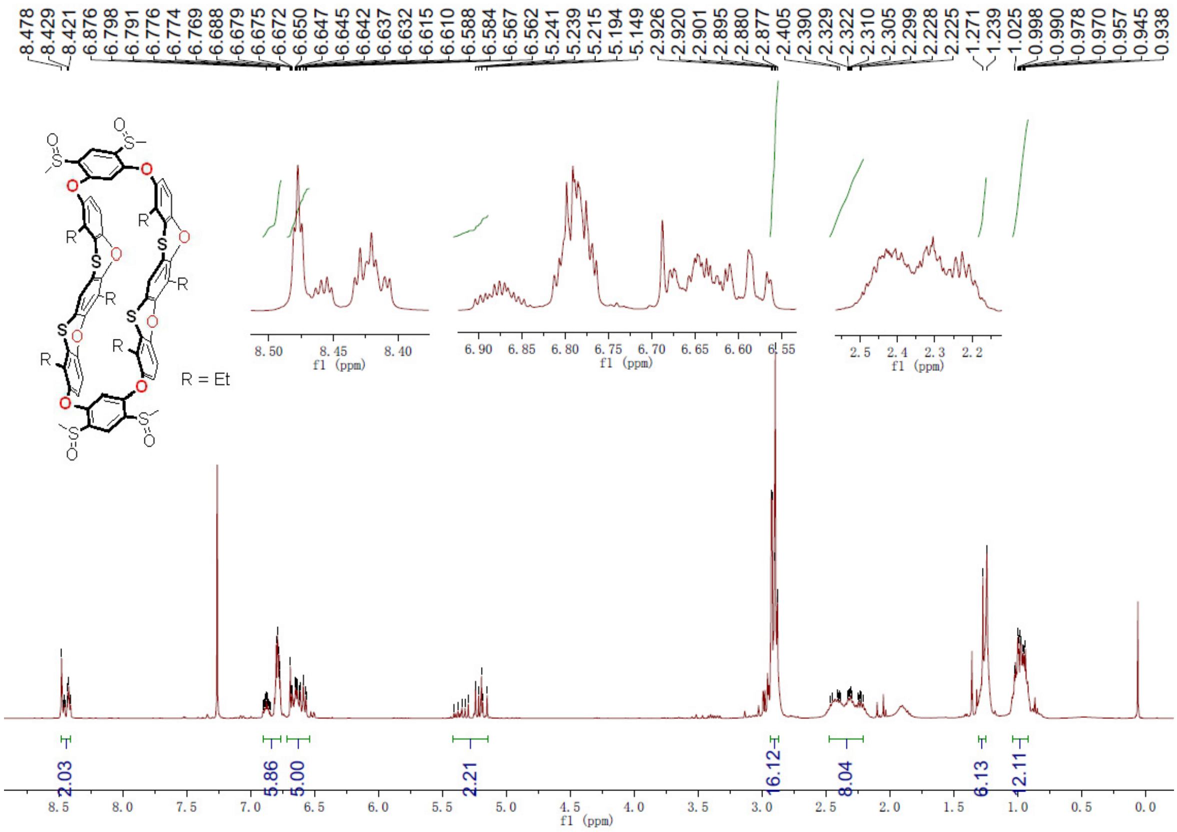

Supplementary Figure 39. ^{1}H NMR Spectrum (400 MHz, $CDCl_3$, 298 K) of **2′**.

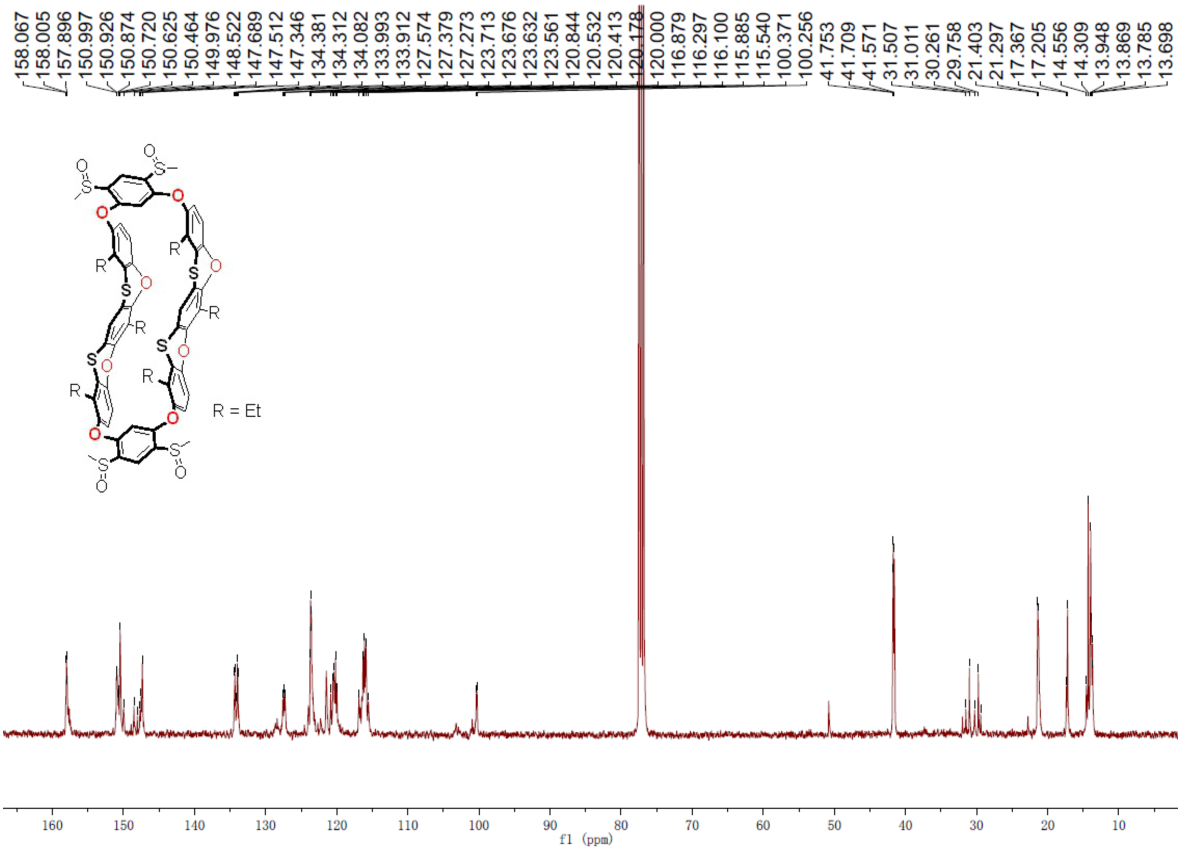

Supplementary Figure 40. ^{1}H NMR Spectrum (400 MHz, $CDCl_3$, 298 K) of **2**.

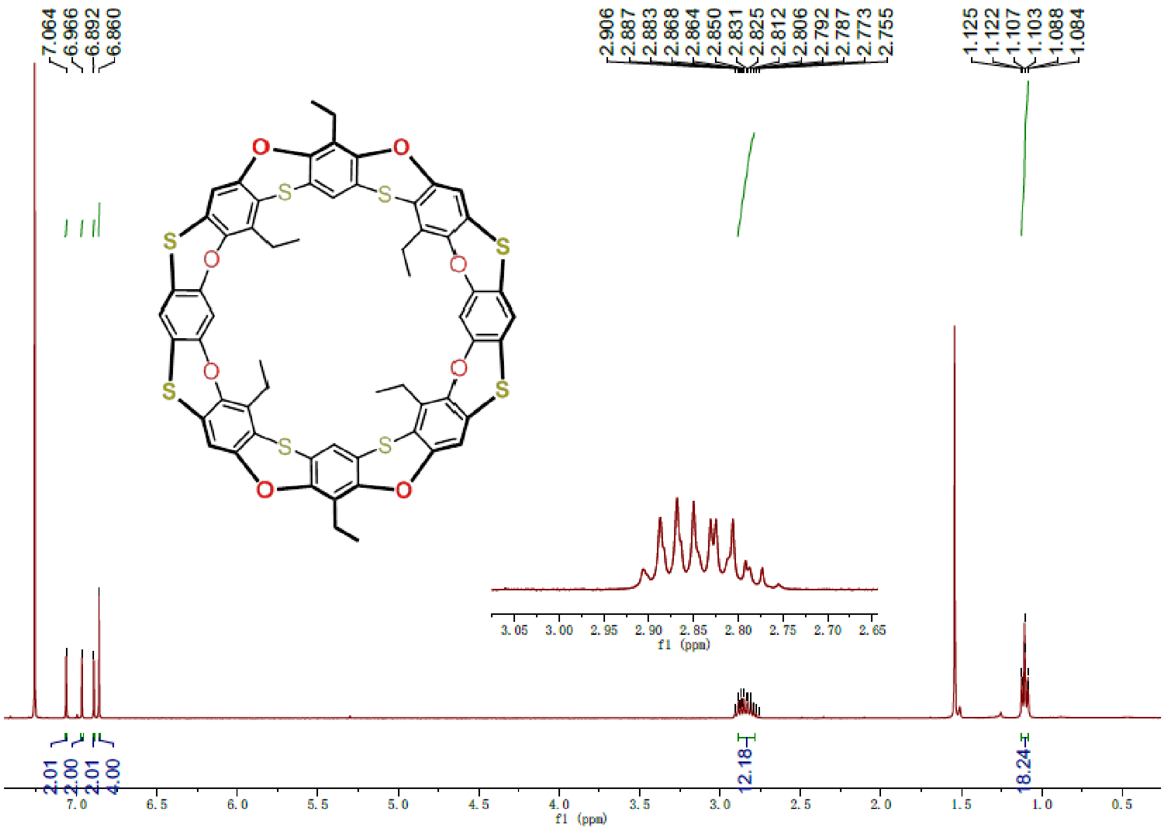

Supplementary Figure 41. ^{1}H NMR Spectrum (400 MHz, $CDCl_3$, 298 K) of **2**.

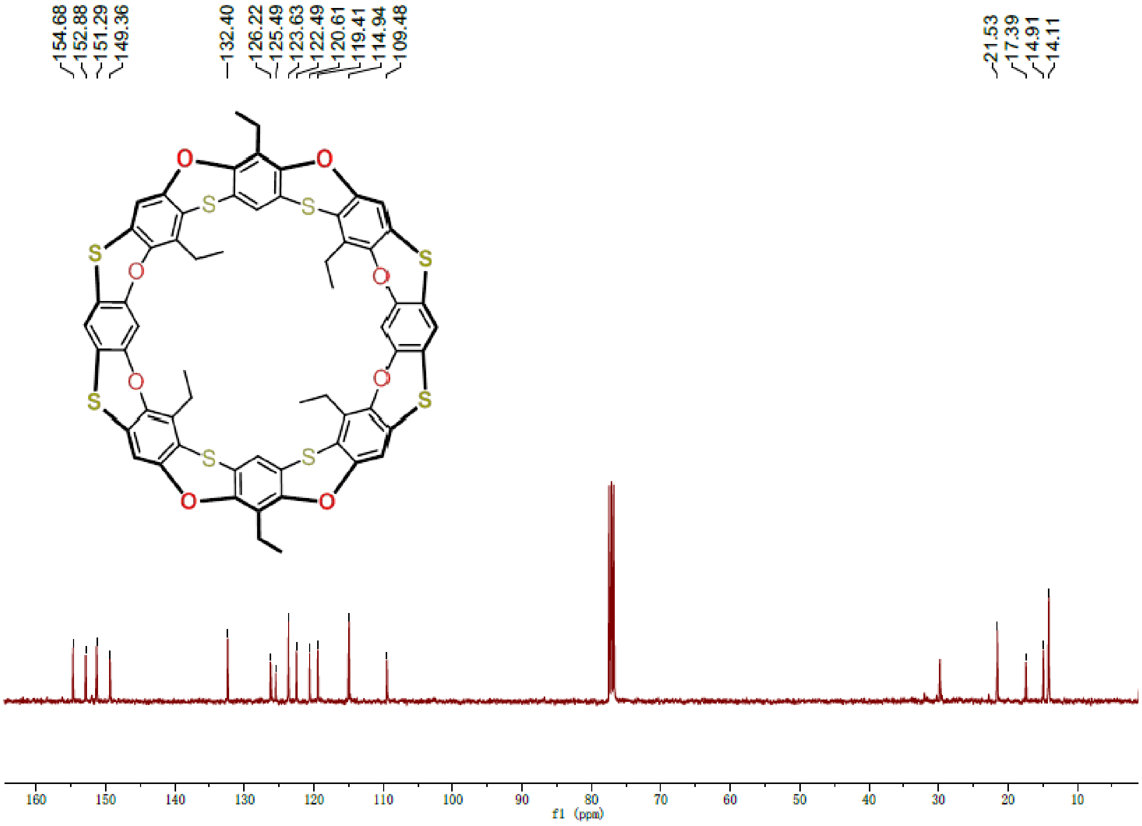

Supplementary Figure 42. ^{13}C NMR Spectrum (100 MHz, CDCl$_3$, 298 K) of **2**.

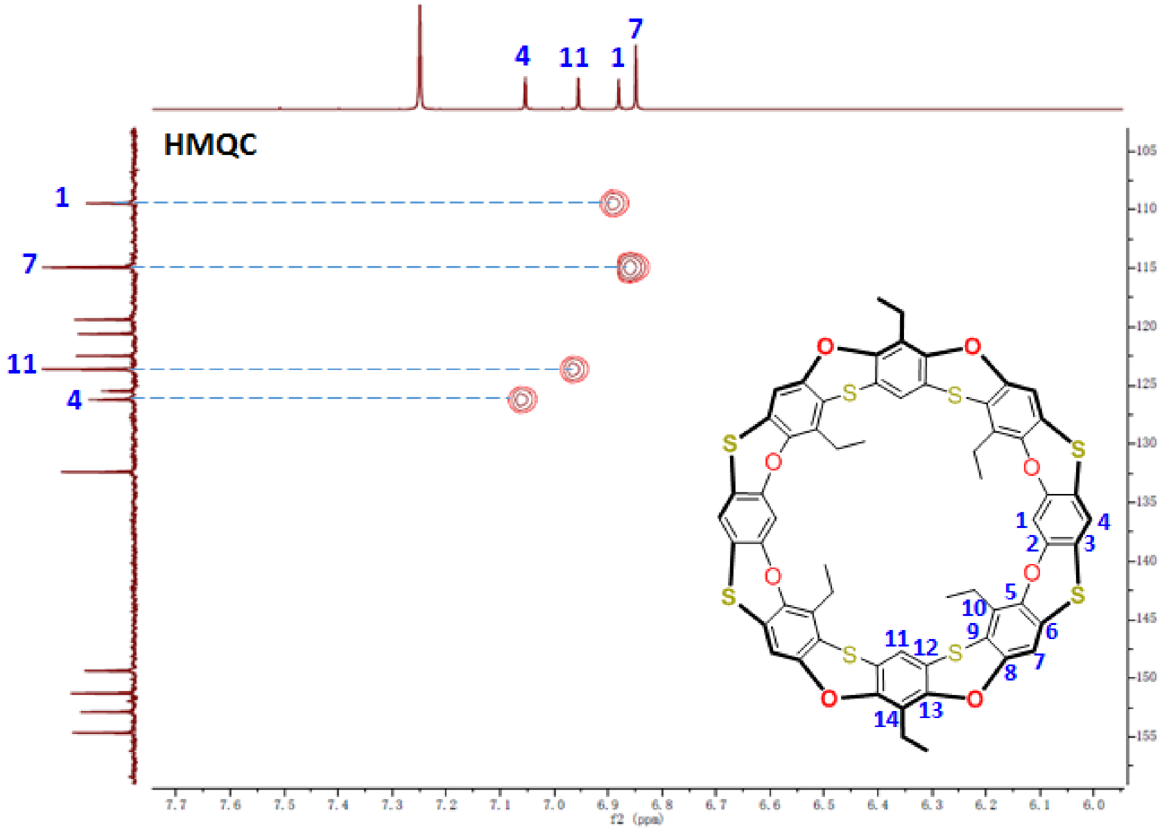

Supplementary Figure 43. ^{1}H-^{13}C HMQC NMR Spectrum (CDCl$_3$, 298 K) of **2**.

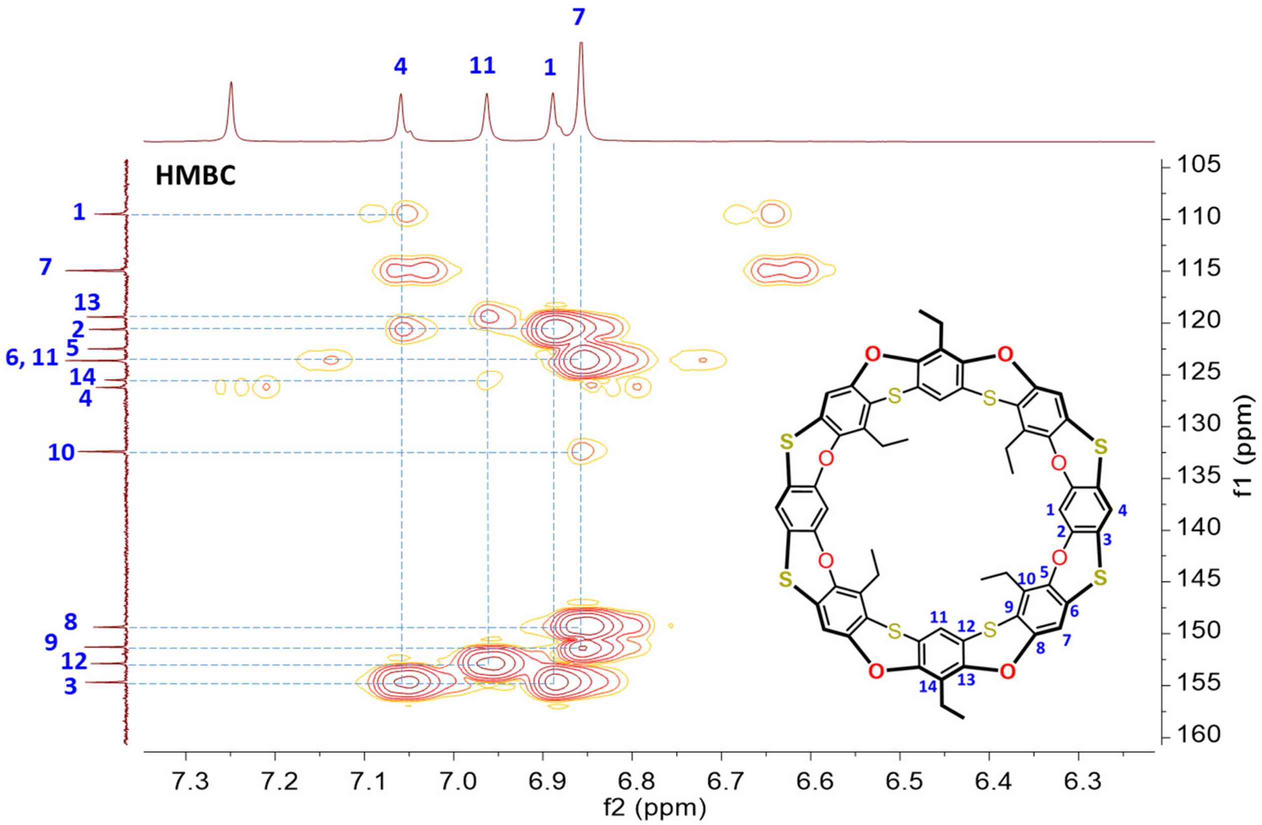

Supplementary Figure 44. ¹H-¹³C HMBC NMR Spectrum ($CDCl_3$, 298 K) of **2**.

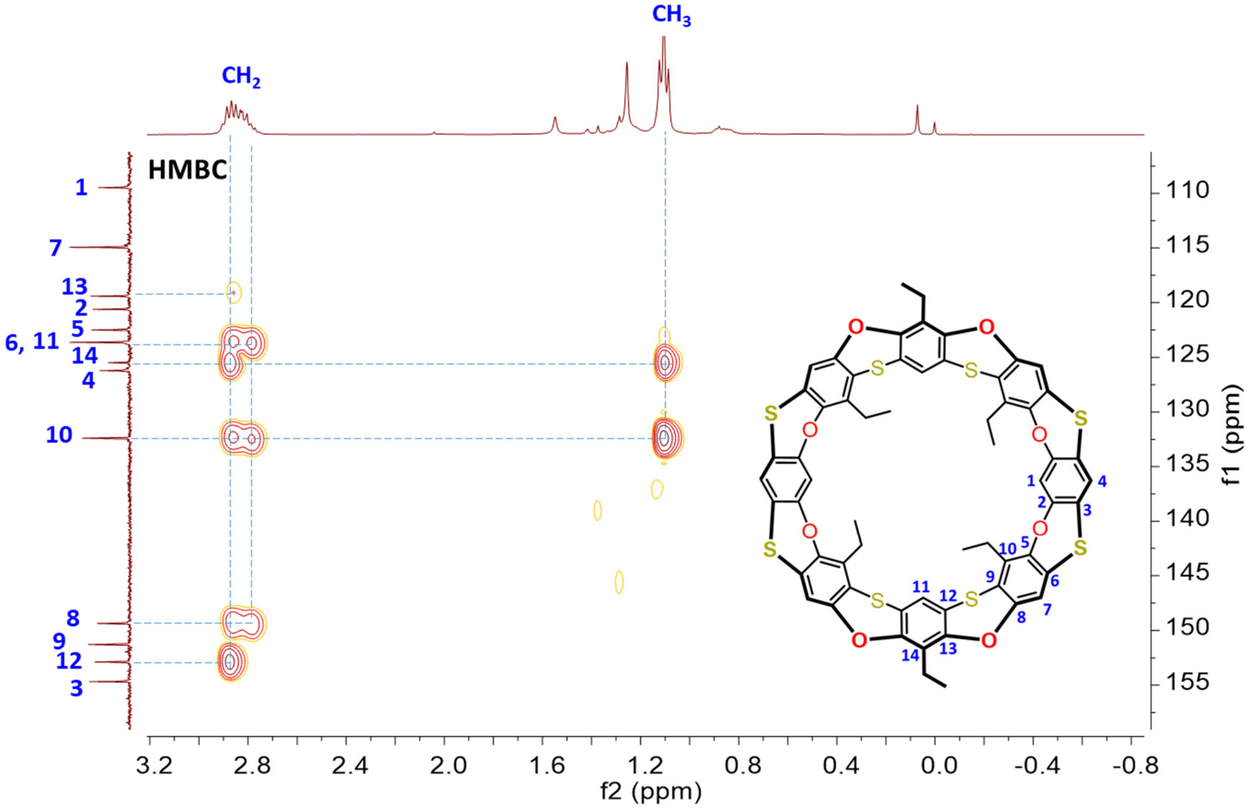

Supplementary Figure 45. ¹H-¹³C HMBC NMR Spectrum ($CDCl_3$, 298 K) of **2**.

Supplementary References

1. Azzena, U. et al. Regioselectivity in the reductive cleavage of pyrogallol derivatives: reductive electrophilic substitution of acetals of 2,3-dimethoxyphenol. *J. Chem. Soc. Perk. T, 1*, **3**, 261−266 (1995).

2. Leuninger, J. et al. Novel Approach to Ladder-Type Polymers: Polydithiathianthrene via the Intramolecular Acid-Induced Cyclization of Methylsulfinyl-Substituted Poly(meta-phenylene sulfide). *Macromol. Chem. Phys.* **202**, 2832−2842 (2001).

3. Oyaizu, K. et al. Synthetic Routes to Polyheteroacenes: Characterization of a Heterocyclic Ladder Polymer Containing Phenoxathiinium-type Building Blocks. *Macromolecules* **35**, 67−78 (2002).

4. Thordarson, K., Bindfit, http://app.supramolecular.org/bindfit/

5. Hibbert, D. B. & Thordarson, P. The death of the Job plot, transparency, open science and online tools, uncertainty estimation methods and other developments in supramolecular chemistry data analysis *Chem. Commun.* **52**, 12792−12805 (2016).

6. Dolomanov, O. V. et al. OLEX2: a complete structure solution, refinement and analysis program. *J. Appl. Cryst.* **42**, 339−341(2009).

7. Sheldrick, G. M. A short history of SHELX. *Acta Cryst. A*, **64**, 112−122 (2008).
